# Mechano-sensitivity of β2-adrenoceptors enhances constitutive activation of cAMP generation that is inhibited by inverse agonists

**DOI:** 10.1038/s42003-024-06128-2

**Published:** 2024-04-05

**Authors:** Sean A. Cullum, Simon Platt, Natasha Dale, Oliver C. Isaac, Edward S. Wragg, Mark Soave, Dmitry B. Veprintsev, Jeanette Woolard, Laura E. Kilpatrick, Stephen J. Hill

**Affiliations:** 1 Division of Physiology, Pharmacology and Neuroscience, School of Life Sciences, University of Nottingham, Nottingham, NG7 2UH UK; 2 Centre of Membrane Proteins and Receptors, University of Nottingham, Nottingham, NG7 2UH UK; 3 Division of Bimolecular Science and Medicinal Chemistry, School of Pharmacy, Biodiscovery Institute, University of Nottingham, Nottingham, NG7 2RD UK

**Keywords:** Receptor pharmacology, Pharmacodynamics

## Abstract

The concept of agonist-independent signalling that can be attenuated by inverse agonists is a fundamental element of the cubic ternary complex model of G protein-coupled receptor (GPCR) activation. This model shows how a GPCR can exist in two conformational states in the absence of ligands; an inactive R state and an active R* state that differ in their affinities for agonists, inverse agonists, and G-protein alpha subunits. The proportion of R* receptors that exist in the absence of agonists determines the level of constitutive receptor activity. In this study we demonstrate that mechanical stimulation can induce β_2_-adrenoceptor agonist-independent Gs-mediated cAMP signalling that is sensitive to inhibition by inverse agonists such as ICI-118551 and propranolol. The size of the mechano-sensitive response is dependent on the cell surface receptor expression level in HEK293G cells, is still observed in a ligand-binding deficient D113A mutant β_2_-adrenoceptor and can be attenuated by site-directed mutagenesis of the extracellular N-glycosylation sites on the N-terminus and second extracellular loop of the β_2_-adrenoceptor. Similar mechano-sensitive agonist-independent responses are observed in HEK293G cells overexpressing the A_2A_-adenosine receptor. These data provide new insights into how agonist-independent constitutive receptor activity can be enhanced by mechanical stimulation and regulated by inverse agonists.

## Abstract (web summary)

Use of a cAMP biosensor to study real-time mechano-sensitive enhancement of β2-adrenoceptor constitutive activity and its inhibition by β2-inverse agonists in HEK 293 cells.

## Introduction

The β_2_-adrenoceptor (β_2_AR) is a class A G protein-coupled receptor (GPCR) that is expressed in airway and vascular smooth muscle, endothelial cells, inflammatory cells and the heart^1–6^. It is an important drug target and β_2_-agonists have been used successfully to treat asthma and chronic obstructive pulmonary disease^7,8^. More recently, there has been interest in the use of β_2_AR antagonists to prevent tumour cell metastasis in breast cancer^9,10^. The primary signalling mechanism by which the β_2_AR mediates intracellular signalling is via coupling to the heterotrimeric Gs protein and the subsequent activation of adenylate cyclase leading to the formation of intracellular cyclic adenosine monophosphate (cAMP)^11,12^.

**Fig. 1:**
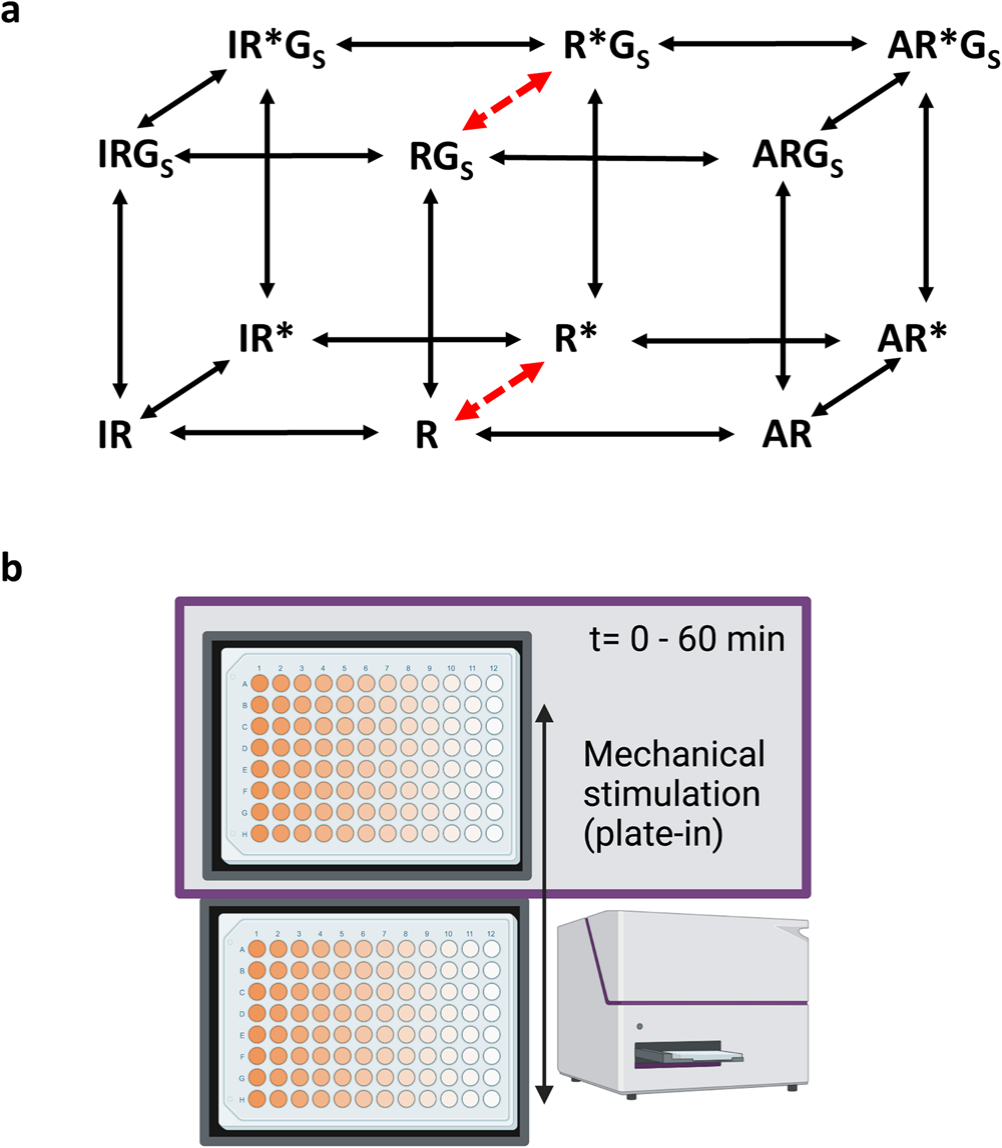
Constitutive Gs-coupled GPCR activity and mechanical stimulation. **a** The cubic ternary complex model for a GPCR interacting with agonists (A), inverse agonists (I) and Gs alpha subunits (Gs). In this model, the receptor can exist in two conformational states in the absence of ligands; an inactive R state and an active R* state that differ in their affinities for agonists, inverse agonists, and Gs alpha subunits. The conformational equilibrium that exists between R and R* (red arrows) explains the concept of constitutive receptor activity whereby basal activity can be observed in the absence of agonists in cells overexpressing native or constitutively active mutant β_2_ARs, as a consequence of binding to Gs proteins. **b** The mechanical mechanism for loading the multi-well plate into the PheraStar plate reader. The 96-well plate is placed on the mechanical plate stage and then automatically taken into the plate reader with a simple motor-driven linear movement. This process has the potential to provide a linear mechanical stimulation of the cell monolayer as the plate enters the light-tight reader. The initial entry of the plate into the reader is normally sufficient to activate the receptor. In most experiments, an initial read was taken at time zero and then the plate was immediately removed for the addition of ligands before re-entering the reader. In some experiments, the plate was added slowly manually to gain an initial GloSensor^TM^ reading of basal levels. Created with Biorender.com.

The mechanisms involved in the activation of signalling following agonist binding are best described by the cubic ternary complex model (Fig. 1a^13–15^). In this model the receptor can exist in two conformational states in the absence of ligands; an inactive R state and an active R* state that differ in their affinities for agonists, inverse agonists, and Gs alpha subunits (Fig. 1). The conformational equilibrium that exists between R and R* explains the concept of constitutive receptor activity whereby basal activity can be observed in the absence of agonists in cells overexpressing native or constitutively active mutant β_2_ARs^16–18^, but also to a lesser extent in native cells expressing the endogenous β_2_AR^19^. In the context of cell lines overexpressing the β_2_AR, there is also the suggestion that receptor dimerisation can contribute to increased constitutive receptor activity ^20^. The basal constitutive receptor activity can be further enhanced by agonists and inhibited by inverse agonists such as ICI-118551^19,21–23^. Single-molecule studies of the β_2_AR have shown that in phospholipid nanodiscs, individual β_2_AR molecules undergo spontaneous transitions between R and R* conformational states^24^ and that the inverse agonist ICI-118551 increases the frequency of deactivation transitions^24^.

The structure of the β_2_AR has been determined in a variety of conformational states which support many features of the cubic ternary complex. These include the receptor bound to a full agonist^25,26^, the partial agonist salmeterol^27^, an inverse agonist^28^ and the Gs protein^11^. Furthermore, studies of purified β_2_AR reconstituted in high-density lipoparticles have revealed that different phospholipids can alter the equilibrium between the active (R*) and inactive (R) states of the receptor^29^. This suggests that constitutive receptor activity is dependent upon the local microenvironment of the receptor.

The β_2_AR is subject to N-glycosylation post-translational modification on the N-terminus (Asn6, Asn15) and second extracellular loop (Asn187^30–32^). The presence of these extracellular β_2_AR-associated glycan chains has been proposed as a mechanism by which β_2_ARs can be activated by pathogens (e.g. *Meningococcal* pili) pulling on these extracellular glycan chains and eliciting mechanical stimulation of the β_2_AR^6,33,34^. These data suggest that mechanical stimulation of the β_2_AR can also alter the equilibrium between R and R* leading to constitutive receptor activation.

We have recently monitored the kinetics of endogenous β_2_AR-mediated cAMP responses in HEK293 cells expressing the cAMP GloSensor^TM^ biosensor^12^. This highly sensitive biosensor consists of a firefly luciferase enzyme genetically fused to the cAMP-binding domain of the protein kinase A (PKA) regulatory subunit (RIIβB) which undergoes a conformational change in the presence of cAMP leading to an increase in luminescence^35^. HEK293 cells endogenously express the β_2_AR at extremely low levels^36^ and in this study, we have exploited the large dynamic range of GloSensor^TM^ to evaluate the real-time kinetics of constitutive cAMP generation and the influence of β_2_AR agonists, inverse agonists and mechanical stimulation on Gs-coupling to cAMP generation in cells expressing recombinant or endogenous β_2_ARs.

## Results

### Agonist-induced β_2_AR responses in HEK293G cells overexpressing the human β_2_-adrenoceptor and the GloSensor^TM^ cAMP biosensor

**Fig. 2:**
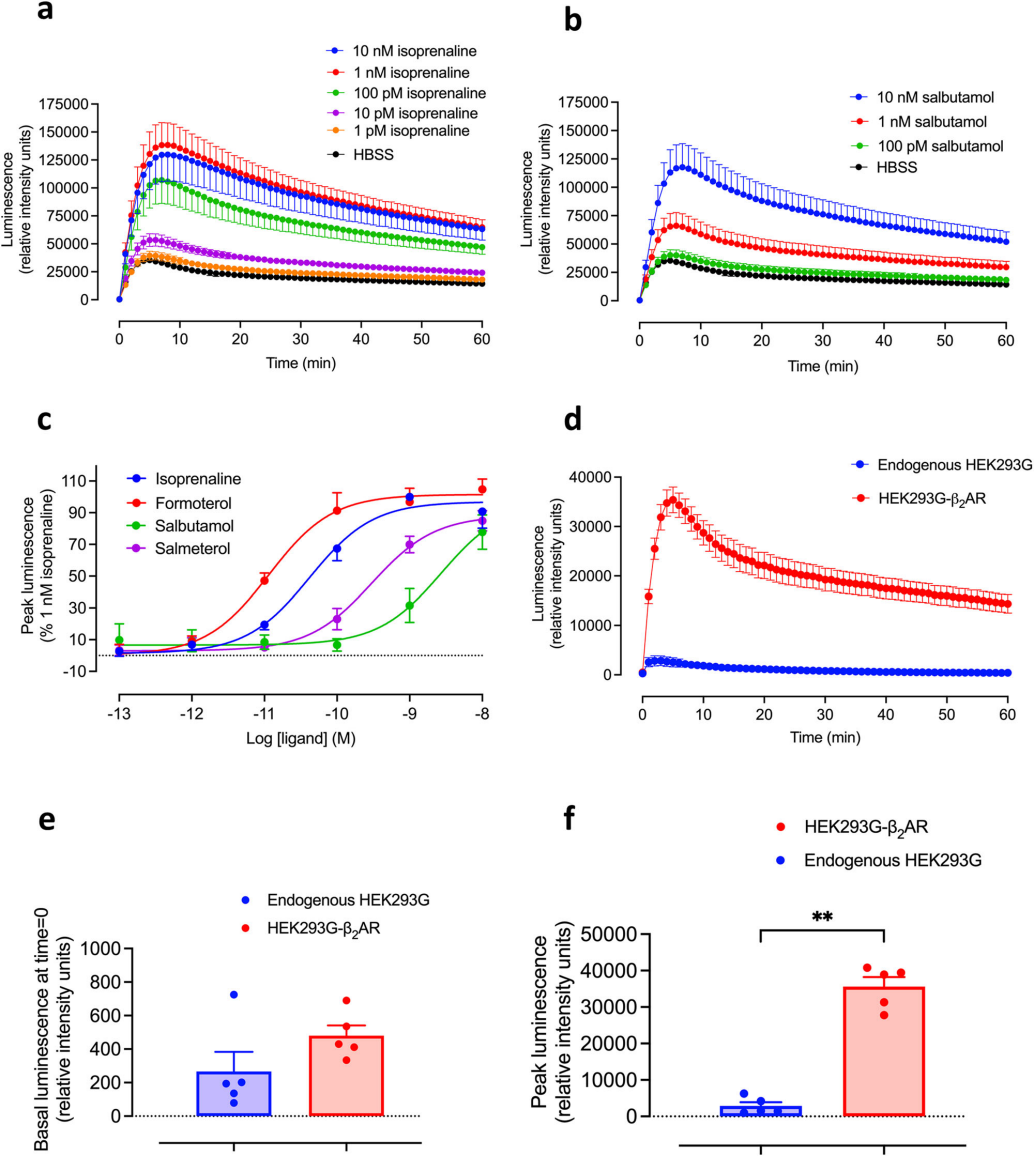
Basal and agonist-induced GloSensor^TM^ luminescence responses in HEK293G cells expressing TS-SNAP-β_2_ARs or endogenous β_2_ARs. Time-course of GloSensor^TM^ luminescence stimulated by increasing concentrations of **a** isoprenaline and **b** salbutamol in a clonal HEK293G cell line overexpressing a transfected human TS-SNAP-β_2_AR. An initial luminescence read was made at time zero. The plate was then immediately removed from the PheraStar, agonists or HBSS added and the plate was then returned to the PheraStar and measurements continued every 1 min for 60 min. Values are mean ± SEM of 5 independent experiments. In each individual experiment, triplicate determinations were made. **c** Concentration–response curves of peak luminescence responses obtained for formoterol, isoprenaline, salmeterol and salbutamol in HEK293G cells overexpressing recombinant TS-SNAP-β_2_AR. Data are expressed as a percentage of the response to 1 nM isoprenaline (after normalisation of HBSS control response to zero) obtained in each individual experiment and represent mean ± SEM from five independent experiments (*n* = 5). **d** A comparison of the time-course of GloSensor^TM^ basal responses to HBSS addition in HEK293G cells overexpressing recombinant TS-SNAP-β_2_AR or wild-type (WT) HEK293G cells expressing endogenous β_2_ARs at very low levels. Values are mean ± SEM from five independent experiments. **e**, **f** Data from **d** showing the response in each cell line at *t* = 0 min prior to the addition of HBSS (**e**) and at the peak of the response to HBSS (**f**). **p = 0.0079 (Mann–Whitney *U* test). At *t* = 0, there was no significant difference between the two cell lines (*p* = 0.15; Mann–Whitney *U* test). Outlier analysis (both ROUT and Grubs method) confirmed that there were no significant outliers in the data sets. It should be noted that in these experiments, the zero time points were taken following initial plate loading and the plate was then immediately removed for the addition of an agonist or HBSS, and the plate was then reinserted into the plate reader.

Figure 2 shows the time courses of GloSensor^TM^ luminescence responses stimulated by increasing concentrations of isoprenaline (Fig. 2a) and salbutamol (Fig. 2b) in a clonal HEK293G cell line overexpressing the human β_2_-adrenoceptor containing an N-terminal Twin-Strep affinity purification tag and an N-terminal SNAP-tag (TS-SNAP-β_2_AR^37^). Peak responses were obtained between 5- and 10-min following agonist addition and then gradually fell back toward basal levels over 60 min. Analysis of the concentration–response characteristics of four different β_2_-agonists (isoprenaline, formoterol, salbutamol, and salmeterol; Fig. 2c; Table 1) indicated that EC_50_ values were much lower than those obtained previously for the endogenous β_2_AR expressed in HEK293G cells, consistent with the expected large increase in spare receptor reserve in the recombinant cell line (Table 1^12^). For example, the EC_50_ for isoprenaline in TS-SNAP-β_2_AR-expressing cells was two orders of magnitude lower than that obtained in the native HEK293G cell line (Table 1). In keeping with this, the relative maximum responses (relative to the full agonist isoprenaline) obtained with the lower efficacy agonists salmeterol and salbutamol were much larger than those obtained in cells endogenously expressing the native β_2_AR (Fig. 2c; Table 1). However, a notable feature of the kinetic profiles shown in Fig. 2a and b is that the basal response to vehicle (HBSS) was significant, reaching a peak over 4–5 min. Over the five experiments shown in Fig. 2a, the peak basal (HBSS) response was 26.9 ± 2.9% of the maximum response obtained with 1 nM isoprenaline in the same experiment (*n* = 5).

**Table 1:** Agonist *E*_max_, and log EC_50_ determined for isoprenaline, formoterol, salbutamol and salmeterol from concentration–response curves obtained by cAMP GloSensor^TM^ in HEK293G cells expressing endogenous β_2_ARs or HEK293G cells overexpressing recombinant TS-SNAP-β_2_AR

Agonist	TS-SNAP-β_2_AR Log EC_50_ (M)	TS-SNAP-β_2_AR *E*_MAX_^a^	Endogenous β_2_AR Log EC_50_ (M)^b^	Endogenous β_2_AR *E*_MAX_^b,c^
Isoprenaline	−10.35 ± 0.15	100	−8.01 ± 0.12	100
Formoterol	−10.95 ± 0.12	104.73 ± 6.50	−9.00 ± 0.04	98.38 ± 4.31
Salbutamol	−8.46 ± 0.16	77.81 ± 10.84	−6.73 ± 0.01	44.74 ± 3.80
Salmeterol	−9.53 ± 0.10	84.94 ± 6.38	−8.39 ± 0.12	33.73 ± 3.60

Values are mean ± SEM from five independent experiments.

^a^EMAX values expressed as a % of the response to 1 nM isoprenaline.

^b^Data taken from ref. ^12^.

^c^EMAX values expressed as a % of the response to 1 μM isoprenaline.

### Comparison of basal GloSensor^TM^ cAMP responses in HEK293G cells expressing recombinant or endogenous β_2_ARs

HEK293G cells are known to express endogenous β_2_ARs at extremely low levels^36,38^, but are nevertheless capable of eliciting strong GloSensor^TM^ cAMP responses in response to β_2_-agonists^12^. We have therefore taken advantage of this to compare the basal HBSS responses in a clonal cell line overexpressing recombinant TS-SNAP-β_2_ARs with that of wild-type HEK293G cells expressing just endogenous receptors (Fig. 2d). What is clear from these data is that the peak basal responses in the two cell lines are dramatically different. This is indicative of constitutive β_2_AR activity in the cell line overexpressing the TS-SNAP-β_2_AR. However, it is noticeable that there was no significant difference in the basal responses measured at time zero between the two cell lines (Fig. 2e), suggesting that the basal response differences develop during the first 5 min of the measurement period (Fig. 2f). These data indicate that the different basal responses are not necessarily due to inherent differences in constitutive activity but are rather a consequence of the significant difference (*p* < 0.0001, unpaired *t*-test) in the generated peak response (Fig. 2f) that follows the mechanical loading of the multi-well plate into the plate reader (Fig. 1b). This suggests that a large proportion of the basal cAMP response is a consequence of mechanical stimulation. This did not appear to be due to the addition of HBSS since a similar large basal response was obtained with no HBSS addition in a clonal cell line overexpressing recombinant TS-SNAP-β_2_ARs (Supplementary Fig. 1).

### Influence of β_2_-inverse agonists on the basal cAMP response

**Fig. 3:**
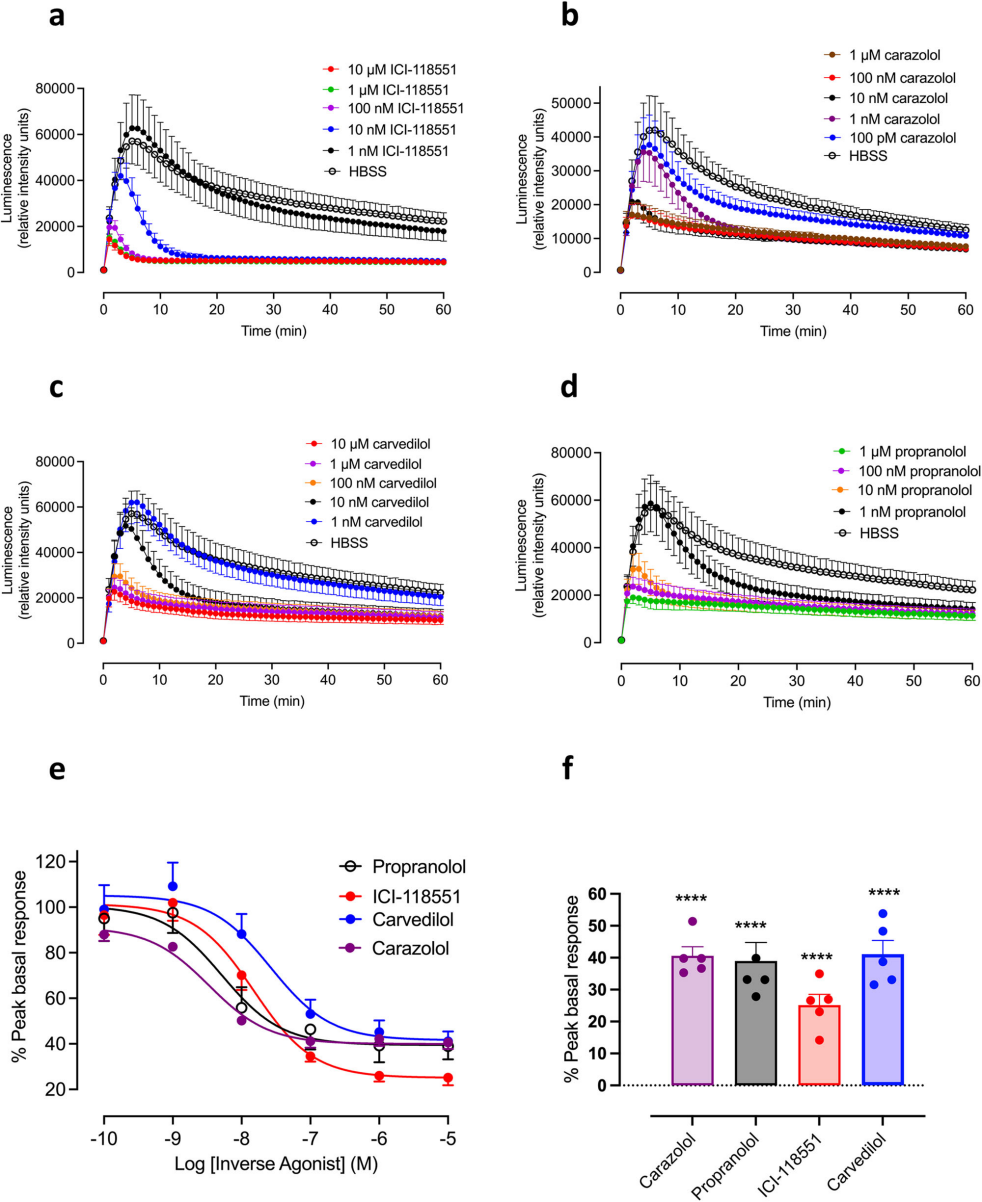
Time-course of the effect of increasing concentrations of β_2_AR inverse agonists on basal-stimulated GloSensor^TM^ responses in a clonal HEK293G cell line overexpressing recombinant TS-SNAP-β_2_AR. **a** ICI-118551; **b** carazolol; **c** carvedilol and **d** propranolol. Each inverse agonist was added immediately following an initial luminescence read at time zero. The plate therefore entered the PheraStar at time zero, was then removed for the addition of inverse agonist or HBSS and measurements were made again at *t* = 1 min and every minute thereafter. Values are mean ± SEM of five independent experiments. In each individual experiment, triplicate determinations were made. **e** Effect of four inverse agonists on the peak basal response to HBSS in a HEK293G clonal cell line over-expressing the transfected human TS-SNAP-β_2_AR. Values are mean ± SEM from five independent experiments. Data are expressed as a percentage of the peak basal response obtained in each individual experiment. **f** Maximal peak inverse agonist responses were obtained in the presence of 10 μM inverse agonist. Data are expressed as a percentage of the peak basal response obtained in each individual experiment. ****P < 0.0001 compared to the peak basal response in the same experiment (paired *t*-test; *n* = 4 or 5).

Four inverse agonists were evaluated as inhibitors of the basal GloSensor^TM^ response in HEK293G cells overexpressing the TS-SNAP-β_2_AR (Fig. 3). Figure 3a shows the effect of increasing concentrations of the most efficacious inverse agonist ICI-118551 on the basal response, when added immediately after an initial luminescence read at time zero. It should be noted that the plate was mechanically loaded into the plate-reader for this initial read and then the plate was removed for the addition of inverse agonist and subsequent re-loading of the plate into the plate-reader. What is clear from the kinetic profiles is that there is an overshoot of the basal response before the inhibitory effect of ICI-118551 is firmly established (usually after 5–10 min). As the concentration of ICI-118551 increases, the peak basal response is markedly reduced and the reduced basal signal is more rapidly achieved (Fig. 3a). Similar effects were seen with carazolol (Fig. 3b), carvedilol (Fig. 3c) and propranolol (Fig. 3d), although there were small differences in the size of the maximal inhibition achieved (Fig. 3e, f). Concentration–response curves for all four inverse agonists are shown in Fig. 3e for their attenuation of the peak basal response to HBSS when both are added together. Log IC_50_ values from the five replicate experiments are shown in Supplementary Table 1 for each inverse agonist. Figure 3f shows the extent of the attenuation of the basal response obtained at 10 μM of each inverse agonist. All responses were significantly lower than the corresponding control basal response (*p* < 0.0001; paired *t*-test). Mean values for the inhibition by 10 μM inverse agonist are also shown in Supplementary Table 1.

**Fig. 4:**
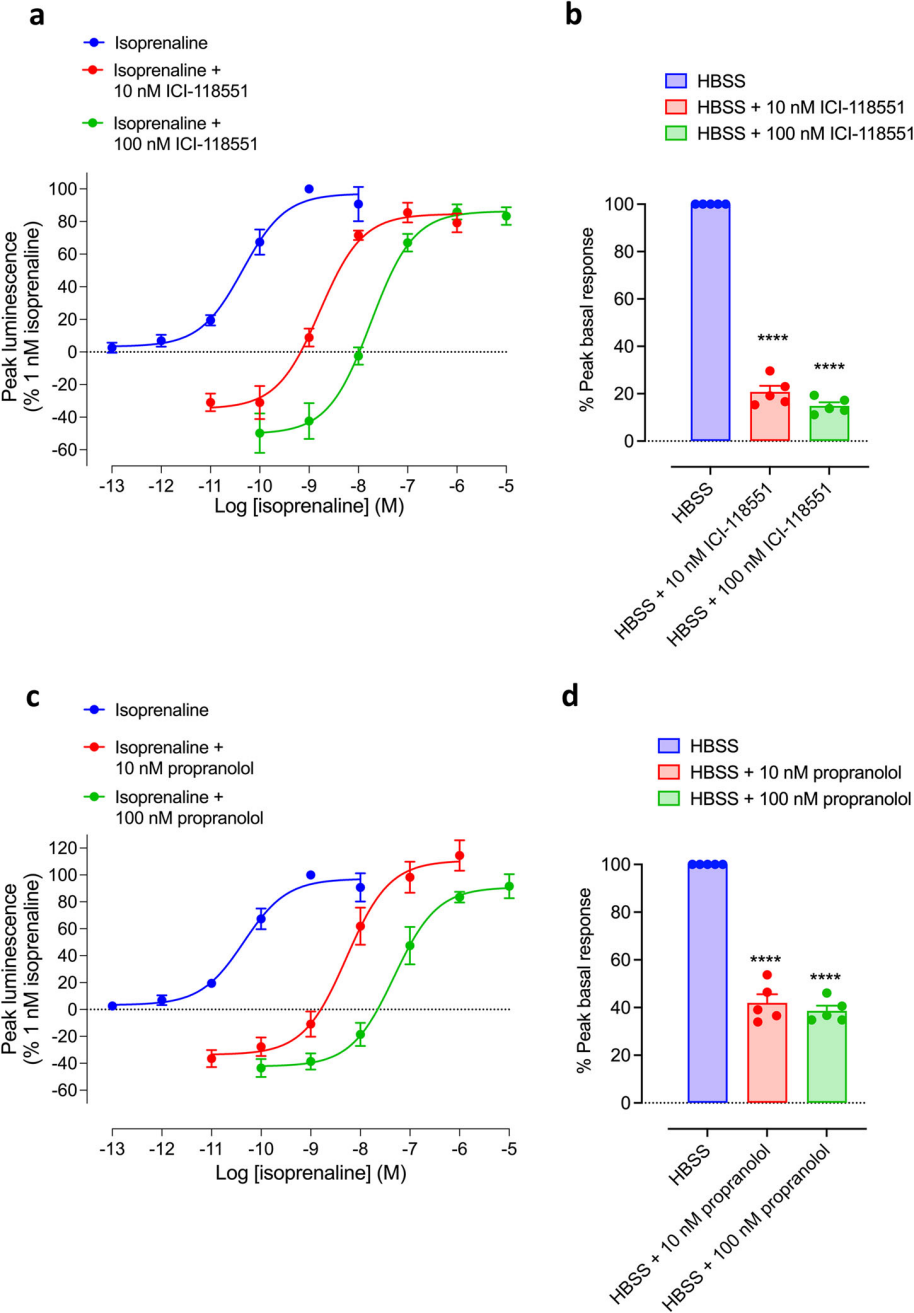
Impact of inverse agonists on agonist-stimulated and basal GloSensor^TM^ responses in HEK293G cells overexpressing recombinant TS-SNAP-β_2_ARs. Concentration–response curves of peak luminescence responses obtained for isoprenaline, in the presence and absence of **a**, **b** 10 nM or 100 nM ICI-118551 or **c**, **d** 10 nM or 100 nM propranolol in HEK293G cells overexpressing recombinant TS-SNAP-β_2_ARs. Inverse agonists were added for 30 min prior to the addition of isoprenaline or HBSS. Data in **a** and **c** are expressed as a percentage of the response to 1 nM isoprenaline (after normalisation of HBSS control response to zero) obtained in each individual experiment and represent mean ± SEM from five independent experiments (*n* = 5). **b**, **d** Peak HBSS responses were obtained in the absence or presence of **b** ICI-118551 or **d** propranolol expressed as a percentage of the HBSS control. ****p < 0.0001 versus HBSS control (paired *t*-test; *n* = 5).

Figure 4 shows the impact of a 30 min preincubation with ICI-118551 or propranolol on the basal and isoprenaline-stimulated responses in HEK293G cells overexpressing the TS-SNAP-β_2_AR. As expected, the inverse agonists produced a large parallel shift of the upper portion of the concentration-response curve to isoprenaline (Fig. 4a, c). However, both effects were accompanied by a significant reduction in the basal responses (Fig. 4b, d) that was overcome by agonist administration, consistent with inverse agonism. The impact of pre-treatment with inverse agonists for 30 min on the log IC_50_ and % maximum inhibition of basal responses is shown in Supplementary Fig. 2 and Supplementary Table 1. The longer incubation with inverse agonists decreased the log IC_50_ values (as expected on reaching equilibrium) and revealed significant differences between the inverse agonist efficacy of ICI-118551 compared to that of the lower efficacy inverse agonist propranolol (in terms of maximum inhibition of basal responses; Supplementary Table 1).

### Impact of sequential mechano-stimulation of HEK293G cells expressing TS-SNAP-β_2_AR on basal responses

**Fig. 5:**
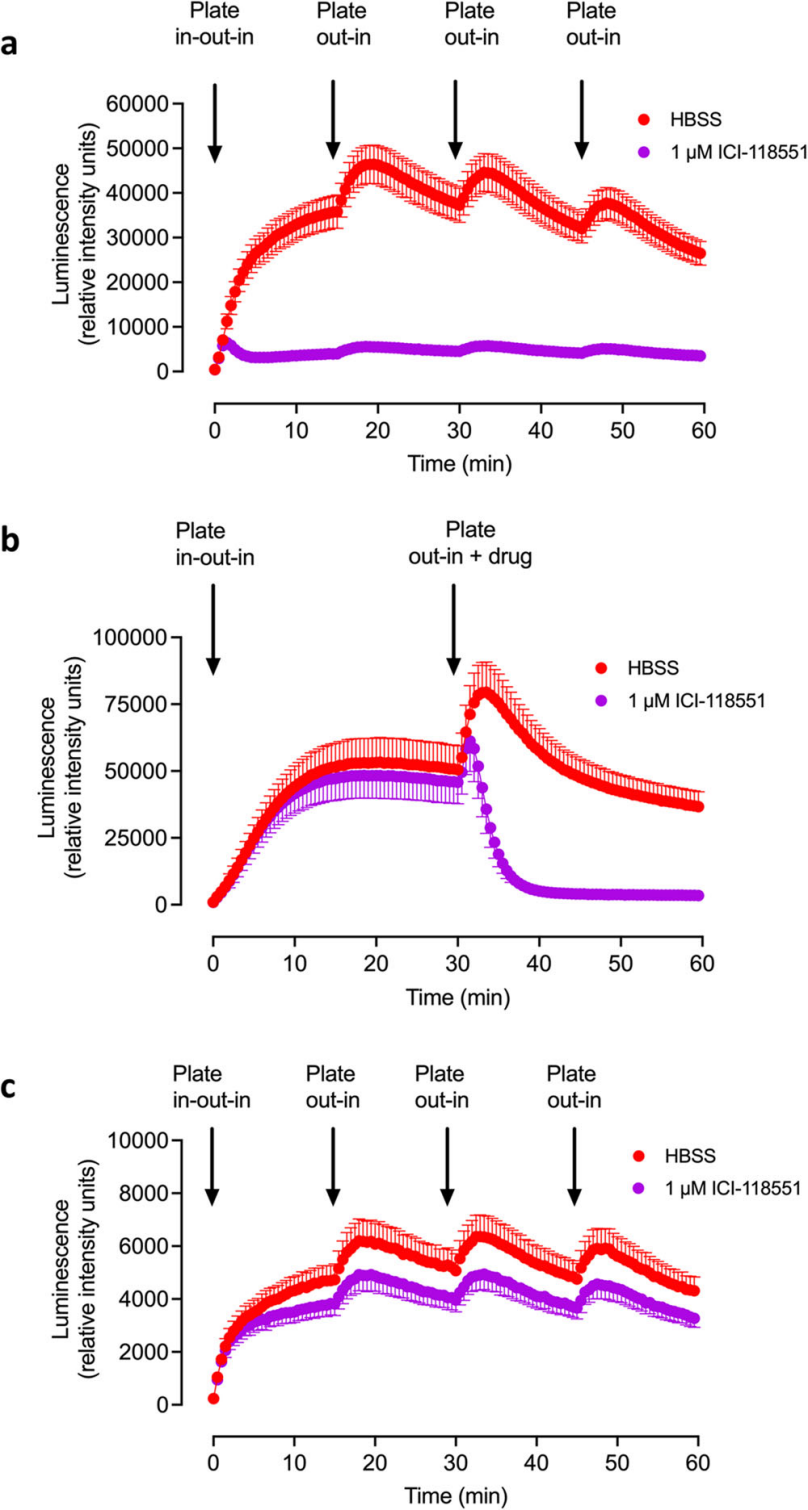
Impact of repeated mechanical stimulation on basal GloSensor^TM^ time-course responses. Impact of mechanical stimulation on basal GloSensor^TM^ time-course responses in a clonal HEK293G cell line overexpressing recombinant TS-SNAP-β_2_AR (**a**, **b**) or HEK293G cells endogenously expressing β_2_ARs (**c**). In all experiments, an initial luminescence read was made at time zero after initial plate entry into the PheraStar and before any additions. The plate was then immediately removed, HBSS or ICI-118551 added, and the plate was then returned to the PheraStar. Measurements were then made at 1 min and every min for 60 min in total. In **a** and **c** HBSS or ICI-118551 (1 μM) were added, and measurements of luminescence continued every minute from time = 1 min. **a**, **c** At 15, 30 and 45 min, the motorised stage of the PheraStar removed the plate from the instrument and then immediately returned it to the plate reader for further measurements every minute. In **b** no additions were made before measurements were made, although the motorised stage removed the plate and returned it to the PheraStar immediately after the time = 0 initial read to be consistent with the experiments in (**a**) and (**c**). At 30 min, the motorised stage of the PheraStar removed the plate from the instrument, HBSS or 1 μM ICI-118551 was added and the plate was then immediately returned to the plate reader for further measurements every min. In **a**–**c** values are mean ± SEM from five independent experiments. In each individual experiment, triplicate determinations were made.

To explore the responses to mechano-stimulation further, we subjected plates containing HEK293G cells over-expressing the TS-SNAP-β_2_AR to consecutive movements of the 96-well plate in and out of the PheraStar reader (Fig. 5). In Fig. 5a, the plate was placed into the PheraStar, and an initial luminescence read was made at time zero, the plate was then immediately removed, HBSS or ICI-118551 (1 μM) added and the plate was then returned to the PheraStar. This led to a large and rapid increase in baseline luminescence that was markedly attenuated by the addition of 1 μM ICI-118551 (Fig. 5a). Removal of the plate, followed by immediate reinsertion into the PheraStar at 15, 30 and 45 min without further additions produced a further increase in basal luminescence that was dramatically reduced by 1 μM ICI-118551 (Fig. 5a). This was not due to light entering the PheraStar during the plate transitions since identical results were obtained when the manipulations were performed in complete darkness (Supplementary Fig. 3). Delaying the addition of HBSS or 1 μM ICI-118551 until 30 min resulted in a secondary increase in luminescence following addition of HBSS, but a rapid decline in luminescence following addition of the inverse agonist (Fig. 5b). This suggested that the response to ongoing mechanical stimulation can be rapidly reversed by inverse agonists (Fig. 5b). The rapid movement of the plate into and out of the PheraStar was essential for mechano-stimulation of the β_2_-adrenoceptor since short linear or orbital movements of the plate within the PheraStar (100 rpm; 5 s) were not sufficient to trigger further increases in basal responses (Supplementary Fig. 4).

Similar results were obtained in HEK293G cells only expressing endogenous β_2_-adrenoceptors at low levels^36,38^, although the size of the mechanical responses obtained was very much lower (Fig. 5c). The mechano-sensitive responses in these cells were also much less sensitive to inhibition by the inverse agonist ICI-118551 (Fig. 5c) indicating that other Gs-coupled GPCRs endogenously expressed in these cells may be also contribute to the basal mechano-stimulatory response (see below).

### Impact of a binding deficient D113A mutation of the human β2-adrenoceptor on basal GloSensor^TM^ cAMP responses in HEK293G cells

**Fig. 6:**
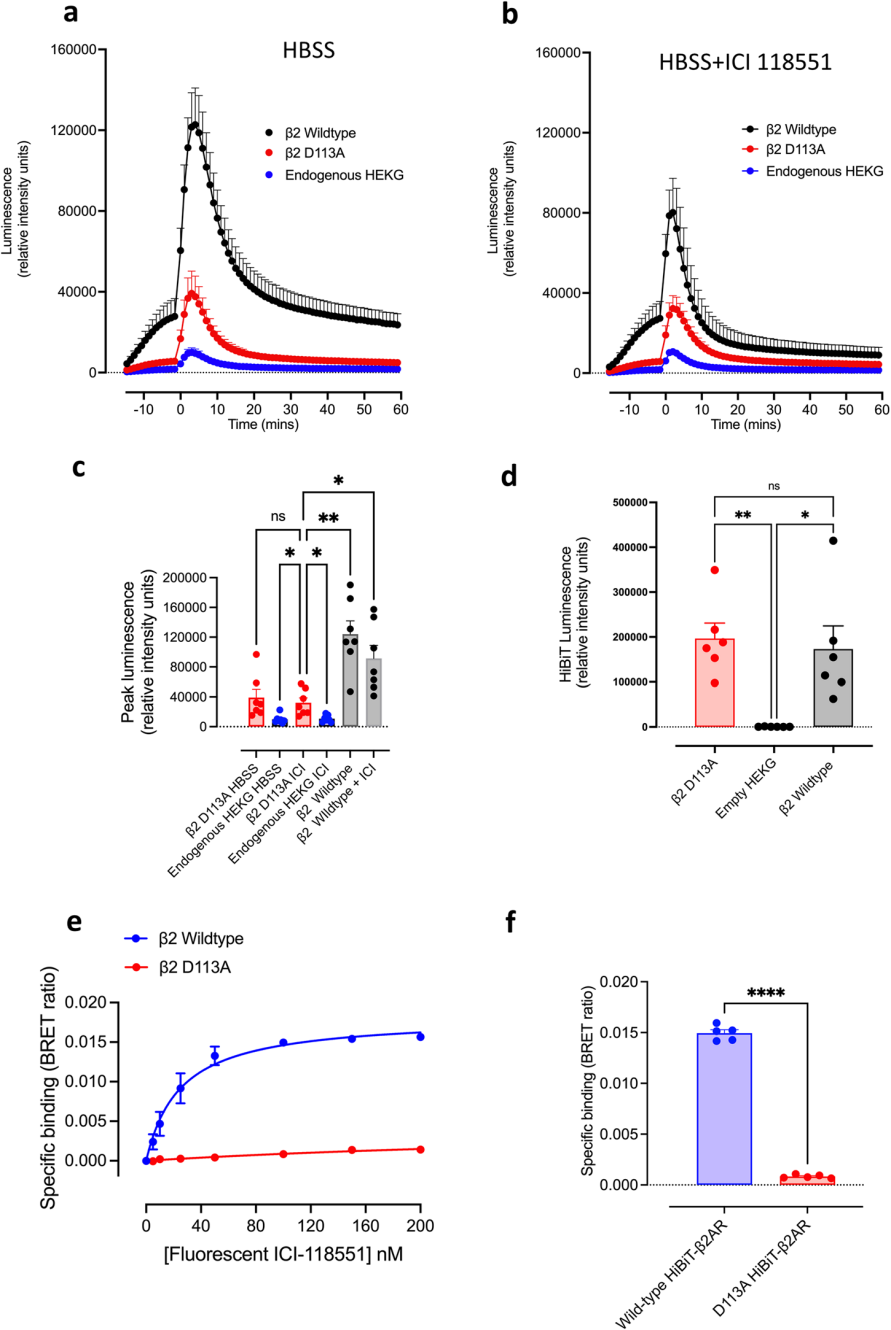
The effect of a binding-deficient mutation (D113A) of the human β_2_-adrenoceptor on the basal mechanical stimulation of GloSensor^TM^ responses obtained in a stable HEK293G cell line over-expressing HiBiT-D113A-β_2_AR. **a**, **b** Time-course of the basal GloSensor^TM^ responses in HEK293G cells expressing HiBiT-D113A-β_2_AR, HEK293G cells expressing HiBiT-β_2_AR wild-type or native HEK293G cells with endogenous-β_2_ARs obtained in the absence (**a**) and presence (**b**) of 1 μM ICI-118551. In both (**a**) and (**b**), the plate was placed in the PheraStar at *t* = −15 min, the plate was then removed at time zero and HBSS or ICI-118551 (1 μM) was added, and the plate immediately returned to the PheraStar. Values are mean ± SEM from seven independent experiments. **c** Comparison of maximal basal peak responses obtained in HEK293G cells over-expressing HiBiT-D113A-β_2_AR, HEK293G cells over-expressing HiBiT-β_2_AR wild-type or in native HEK293G cells with endogenous expression of β_2_ARs. Values are mean ± SEM from seven independent experiments. *p < 0.05 or **p < 0.01 (one-way ANOVA with Holm-Sidak multiple comparison test). HEK293G-HiBiT-D113A-β_2_AR versus HEK293G-HiBiT-D113A-β_2_AR in the presence of 1 μM ICI-118551 (not significant, *p* = 0.32); HEK293G-HiBiT-D113A-β_2_AR plus ICI-118551 versus endogenous HEK293G cells plus or minus ICI-118551 (both *p* = 0.035); HEK293G-HiBiT-D113A-β_2_AR plus 1 μM ICI-118551 versus wild-type β_2_AR (*p* = 0.009). **d** Cell surface expression of HiBiT-D113A-β_2_AR in HEK293G cells. Receptor expression was monitored as reconstituted nanoluciferase luminescence following the addition of 0.2% purified LgBiT and 0.2% furimazine. Values are mean ± SEM from six independent experiments. *p < 0.05 or **p < 0.01 (ANOVA with Tukey’s multiple comparison test for matched data). *P* = 0.045 and 0.005 for HiBiT-β_2_AR and HiBiT-D113A-β_2_AR, respectively relative to untransfected HEK293G cells. **e** Specific binding of ICI-118,551-βAla-βAla-BODIPY-X-630/650 (fluorescent ICI-118551) to HEK293G cells expressing wild-type HiBiT-β_2_AR or HiBiT-D113A-β_2_AR. Total and non-specific binding was determined following the re-complementation of full-length nanoluciferase with the addition of 0.2% purified LgBiT. Non-specific binding was determined in the presence of 50 μM ICI-118551. Specific binding was determined by subtraction of non-specific binding from the total binding at each concentration of fluorescent ICI-118551. Values are mean ± SEM from five independent experiments. **f** Specific binding determined with 100 nM fluorescent ICI-118551 in HEK293G cells expressing wild-type HiBiT-β_2_AR or HiBiT-D113A-β_2_AR. Data taken from (**e**) showing mean ± SEM and the individual means obtained in five separate experiments. ****p < 0.0001 (paired *t*-test).

One possible explanation for the enhanced basal responses in HEK293G cells overexpressing the β_2_-adrenoceptor is that there is a basal release of catecholamines induced by mechanical stimulation from the extracellular matrix (ECM) following earlier sequestration (during growth of the cells) of catecholamines present in the growth medium containing foetal calf serum. With the increased sensitivity of the high-expressing cells to β_2_-agonists, this might explain the exaggerated basal response observed in the recombinant cell line. To test this, we have generated a binding deficient mutant human β_2_-adrenoceptor containing a D113A mutation (HiBiT-D113A-β_2_AR). The D113 residue in transmembrane 3 is essential for binding the amine moiety of catecholamines^26,39,40^. To be able to monitor cell surface expression of this HiBiT-D113A-β_2_AR, an 11 amino acid HiBiT tag was attached to the N-terminus of the β_2_-adrenoceptor^36,41^. Figure 6a shows the basal response to mechanical stimulation in a stable cell line expressing the HiBiT-D113A-β_2_AR. In these cells there is still a marked effect of mechano-stimulation. In these experiments, the plate was put into the PheraStar fifteen minutes before the addition of HBSS at time zero. During this time, there was a significant enhancement of cAMP accumulation over that observed in native HEK293G cells (Fig. 6a). At time zero, the plate was removed, HBSS added, and then returned to the PheraStar. This led to a further enhanced basal response (presumably due to the secondary movement of the plate out and back into the plate reader (Fig. 6a). However, it was notable that the basal response to HBSS addition in HiBiT-D113A-β_2_AR-expressing cells was much lower than that obtained in HiBiT-β_2_AR wild-type cells (Fig. 6a) suggesting that there may be some release of catecholamines induced by mechanical stimulation from the extracellular matrix (ECM) in the cells expressing wild-type HiBiT-β_2_AR.

A similar mechanical effect was seen in cells expressing the HiBiT-D113A-β_2_AR and treated with ICI-118551 (Fig. 6b), consistent with a markedly reduced affinity of ICI-118551 for D113A-β_2_AR. Statistical analysis of the peak responses obtained in HEK293G cells over-expressing D113A-β_2_AR, HiBiT-β_2_AR and in native HEK293G cells expressing endogenous levels of the β_2_-adrenoceptor confirmed significant differences between the peak responses to mechanical stimulation (ANOVA; Fig. 6c) but no significant difference in the peak responses obtained in the absence and presence of 1 μM ICI-118553 in HEK293G cells over-expressing D113A-β_2_AR (Fig. 6c).

To confirm successful expression of the HiBiT-D113A-β_2_AR at the cell surface, cell impermeable LgBiT (0.2%) was applied to cells to reconstitute full-length nanoluciferase^42^ and luminescence was measured following the addition of the nanoluciferase substrate furimazine (Fig. 6d). The ability to reconstitute a full-length nanoluciferase on the N-terminus of wild-type HiBiT-β_2_AR and HiBiT-D113A-β_2_AR by addition of purified LgBiT also allowed us to perform ligand-binding studies with ICI-118551-βAla-βAla-BODIPY-X-630/650^36^ to confirm that the D113A mutation yielded a binding-deficient mutant receptor (Fig. 6e, f). In cells expressing the HiBiT-β_2_AR specific binding of the fluorescent analogue of ICI-118551 was clearly demonstrated (Fig. 6e), yielding a *K*_D_ value of 32.65 ± 7.42 nM (*n* = 5 independent experiments). In marked contrast, no saturable component of specific binding of ICI-118551-βAla-βAla-BODIPY-X-630/650 could be detected in cells expressing the HiBiT-D113A-β_2_AR (Fig. 6e, f). In addition, functional data obtained for cells transfected with a D113A-β_2_AR receptor containing a C-terminal nanoluciferase tag confirmed that it did not respond to isoprenaline (measured by monitoring NanoBRET in HEK293G cells expressing mCherry-tagged nanobody-80; Supplementary Fig. 5).

The use of the small eleven amino acid HiBiT tag also allowed us to investigate whether the mechano-sensitive response was partly a consequence of the 19.4 kDa SNAP-tag present on the N-terminus of the β_2_-adrenoceptor in the TS-SNAP-β_2_AR cell line used in earlier experiments. A large mechano-stimulation was still observed in HiBiT-D113A-β_2_AR cells expressing only the 1.3 kDa 11 amino acid HiBiT tag^42^ on the N-terminus of the β_2_-adrenoceptor (Fig. 6a). To explore this further, we also generated a stable HiBiT-wild-type-β_2_AR cell line and confirmed that a basal response to mechano-stimulation could also be generated in this cell line (Supplementary Fig. 6a). Furthermore, when the full-length nanoluciferase protein was reconstituted on the N-terminus of the HiBiT-wild-type-β_2_AR by addition of the 18.1 kDa LgBiT polypeptide^42^ prior to monitoring mechano-sensitive GloSensor^TM^ responses, there was no significant difference between the mechano-sensitive responses observed with or without prior addition of LgBiT (Supplementary Fig. 6b, c).

### The effect of N-glycosylation mutations of the human β_2_AR on the basal mechanical stimulation of GloSensor^TM^ responses in transiently transfected HEK293G cells

**Fig. 7:**
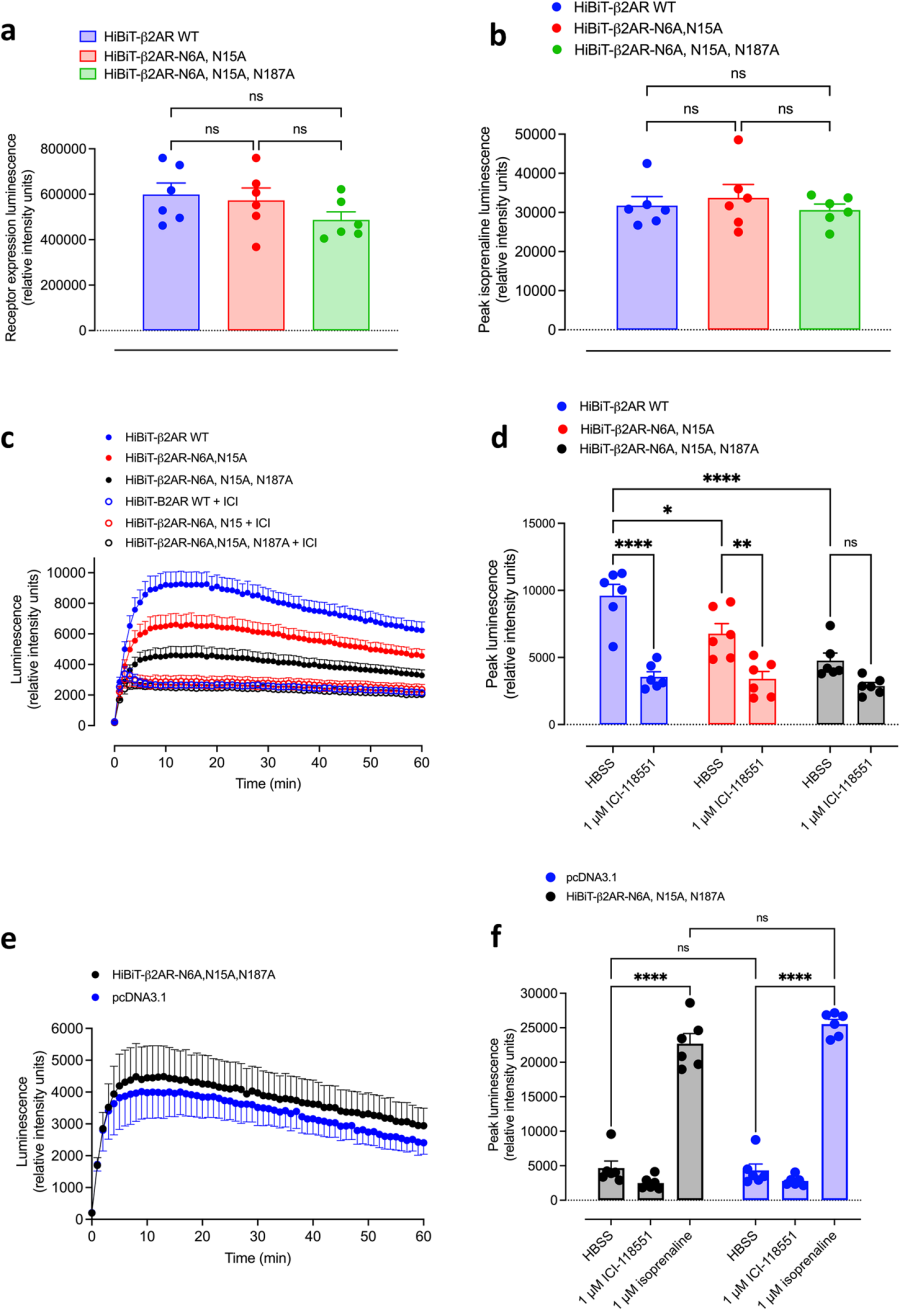
The effect of N-glycosylation mutations of the human β_2_AR on the basal mechanical stimulation of GloSensor^TM^ responses in transiently transfected HEK293G cells. Cells were transiently transfected with the wild-type (WT) β_2_AR, a double mutant (N6A and N15A) β_2_AR or a triple mutant ((N6A, N15A, N178A) β_2_AR or a pcDNA3.1 control. Each β_2_AR construct contained an N-terminal HiBiT sequence. **a** Comparison of the cell surface expression of the three β_2_AR constructs. Receptor expression was monitored as reconstituted nanoluciferase luminescence following the addition of 0.2% purified LgBiT and 0.25% furimazine. Values are mean ± SEM from six independent experiments. There was no significant difference between the expression levels (one-way ANOVA with Tukey’s multiple comparison tests; *p* = 0.923, *p* = 0.252 and *P* = 0.427 for WT versus double mutant, WT versus triple mutant, and double mutant versus triple mutant, respectively). **b** Comparison of maximal peak responses to 1 μM isoprenaline produced by the three β_2_AR constructs. Values are mean ± SEM from six independent experiments. There was no significant difference between the maximal responses (one-way ANOVA with Tukey’s multiple comparisons test; *p* = 0.844, *p* = 0.946 and *P* = 0.663 for WT versus double mutant, WT versus triple mutant and double mutant versus triple mutant, respectively). **c** Time-course and **d** peak basal GloSensor^TM^ responses in the presence and absence of 1 μM ICI-118551. An initial luminescence read was made at time zero. The plate was then immediately removed, HBSS or ICI-118551 added and then the plate was returned to the PheraStar. Measurements were then made at 1 min and every min for 60 min in total. Values are mean ± SEM from six independent experiments. ****p < 0.0001, **p = 0.005; *p = 0.023 (two-way ANOVA with Tukey’s multiple comparison test). 1 μM ICI-118551 had no significant effect on the basal response to the triple mutant (*p* = 0.595). **e** Time-course and **f** peak basal GloSensor^TM^ responses in the presence and absence of 1 μM ICI-118551 or 1 μM isoprenaline **f** in HEK293 cells transfected with the triple β_2_AR mutant or pcDNA3.1. Values are mean ± SEM from six independent experiments. ****p < 0.0001 (two-way ANOVA with Sidak’s multiple comparisons test). The peak responses to isoprenaline were not significantly different (*p* = 0.380). The peak responses to HBSS were also not significantly different (*p* > 0.99). In both the triple mutant (*p* > 0.77) and the pcDNA3.1 control cells (*p* > 0.98), the inhibition by 1 μM ICI-118551 did not reach significance.

As mentioned earlier, the β_2_-adrenoceptor is subject to N-glycosylation on the N-terminus (Asn6, N6; Asn15, N15) and second extracellular loop (Asn187, N187^30–32^). The presence of these extracellular β_2_AR-associated glycan chains has been previously proposed as a mechanism by which β_2_ARs may be activated by pathogens pulling on these extracellular glycan chains and eliciting mechanical stimulation of the β_2_AR^6,33,34^. This mechanical activation has also been demonstrated with beads coated with lectins that recognise sialic acid residues when the beads are submitted to orbital rotation at 100 rpm^33^. To explore the role of β_2_AR glycan chains in the mechano-stimulation of cAMP responses observed in the present study, we investigated the impact on basal GloSensor^TM^ responses of HEK293G cells transiently expressing HiBiT-tagged wild-type β_2_-adrenoceptors (HiBiT-β_2_AR-WT), HiBiT-β_2_AR-N6A-N15A or HiBiT-β_2_AR-N6A-N15A-N187A mutant receptors (Fig. 7). Cell surface expression of the three HiBiT-tagged receptors was not significantly different (Fig. 7a) and they all produced similar GloSensor^TM^ response to 1 μM isoprenaline (Fig. 7b). The time-course of the basal GloSensor^TM^ responses to HBSS addition in cells transiently expressing the three HiBiT-β_2_AR constructs is shown in Fig. 7c. There was a marked and significant attenuation of the peak basal mechanical responses in the cells expressing the HiBiT-β_2_AR-N6A-N15A (*p* = 0.023; two-way ANOVA with Tukey’s multiple comparison test; *n* = 6) or HiBiT-β_2_AR-N6A-N15A-N187A (*P* < 0001) mutant receptors (Fig. 7c, d). For both HiBiT-β_2_AR-WT and HiBiT-β_2_AR-N6A-N15A the response to HBSS was significantly inhibited by 1 μM ICI-118551 (Fig. 7d). In the case of the triple mutant, the inhibition by 1 μM ICI-118551 did not reach significance (Fig. 7d).

To determine whether the mechanical response in the triple mutant cells was significantly different from that obtained with endogenously expressed β_2_-adrenoceptors in HEK293G cells, we directly compared the basal responses to HBSS obtained in HEK293G cells following transient transfection with HiBiT-β_2_AR-N6A-N15A-N187A or the empty transfection vector pcDNA3.1 (Fig. 7e, f). There was no significant difference between them (Fig. 7f). Furthermore, in both the triple mutant and the pcDNA3.1 control cells, the inhibition by 1 μM ICI-118551 did not reach significance (Fig. 7f). These data suggest that other Gs-coupled GPCRs might be contributing to the mechano-sensitive basal responses in native HEK293G cells and in HEK293G cells over-expressing the HiBiT-β_2_AR-N6A-N15A-N187A mutant receptor. Ligand-binding experiments with fluorescent ICI-118551 confirmed that the HiBiT-β_2_AR-N6A-N15A-N187A mutant was able to bind this fluorescent ligand with high affinity at the cell surface (Supplementary Fig. 7). In addition, functional data for the β_2_AR-N6A-N15A-N187A receptor containing a C-terminal nanoluciferase tag confirmed that it responded to isoprenaline with similar pEC_50_ values (8.65 ± 0.11; *n* = 5) to those obtained for wild-type β_2_AR-NanoLuc (8.75 ± 0.04; *n* = 5; measured by monitoring NanoBRET in HEK293G cells expressing mCherry-tagged nanobody-80; Supplementary Fig. 5).

To further explore the basal responses obtained following mechanical stimulation, we investigated the effect of slowly and manually loading plates to obtain a basal GloSensor^TM^ luminescence signal prior to any fast automated linear movements of the plate due to the automated unloading and loading of plates (Supplementary Fig. 8a). These experiments confirmed that the initial basal GloSensor^TM^ signal was not significantly different in HEK293G cells transfected with HiBiT-β_2_AR-N6A-N15A-N187A, HiBiT-β_2_AR or pcDNA3.1 (Supplementary Fig. 8b).

### Role of Gs-coupled adenosine receptors in basal mechano-sensitive responses in HEK293G cells

Adenosine triphosphate (ATP) is known to be released by mechanical forces acting on cultured cells^43,44^. ATP is rapidly metabolised by ectonucleotidases^45,46^ to adenosine, where it can act on Gs-coupled adenosine A_2A_- and A_2B_-receptors^47^. We have previously shown that HEK293G cells express endogenous A_2A_- and A_2B_-receptors that are well coupled to adenylyl cyclase and produce robust GloSensor^TM^ responses^47^. We have therefore investigated the effect of adenosine deaminase on basal GloSensor^TM^ mechanical responses in HEK293G cells.

**Fig. 8:**
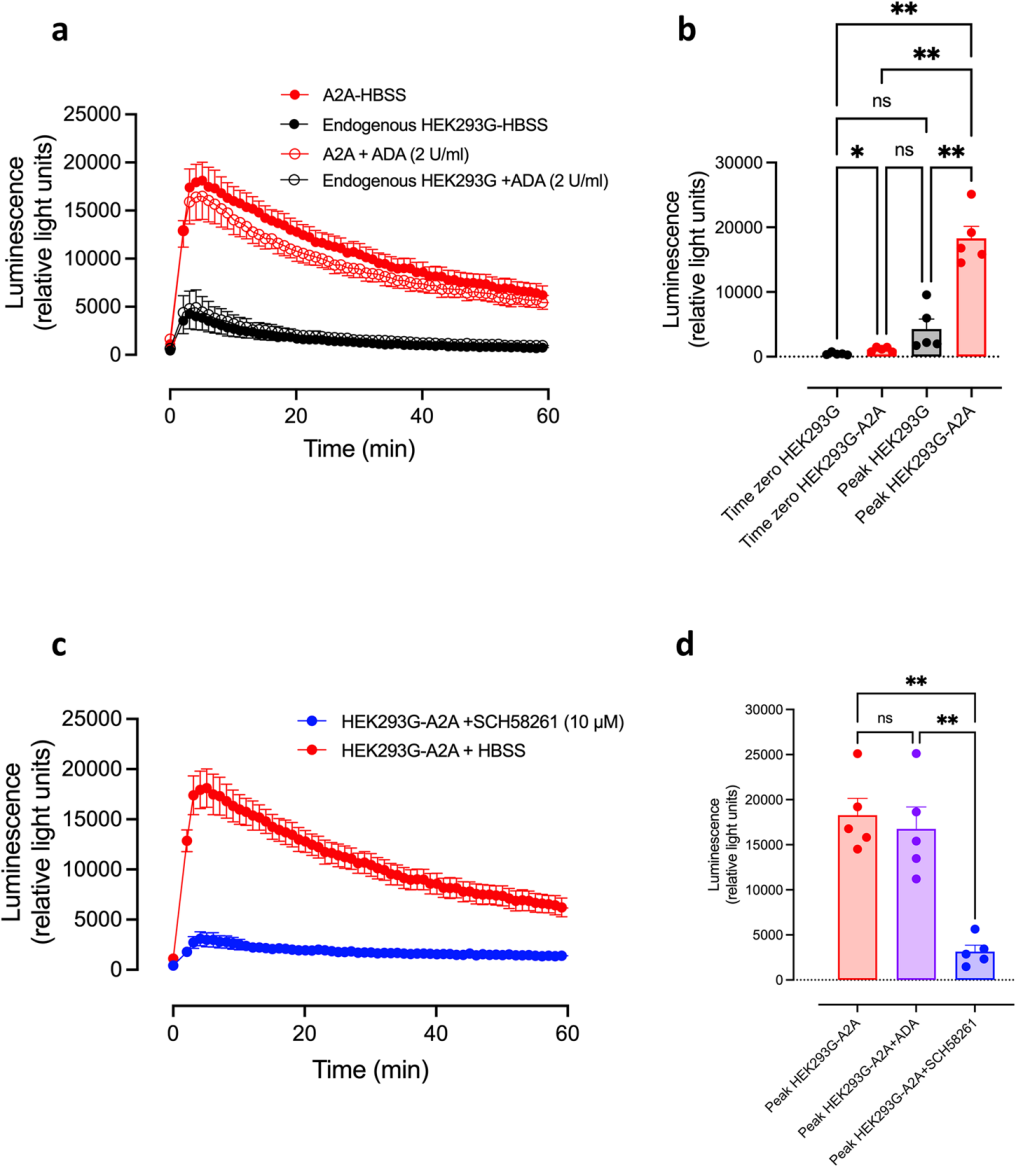
The effect of A_2A_ receptor overexpression on the basal mechanical stimulation of GloSensor^TM^ responses in transiently transfected HEK293G cells. Cells were transiently transfected with cDNA encoding the HiBiT-A_2A_ receptor and studied in comparison to non-transfected cells. Values represent mean ± SEM from five independent experiments. **a** The cAMP response in A_2A_ receptor overexpressing and WT HEK293G cells in the presence or absence of a 2-h pre-treatment with adenosine deaminase (2 U/ml). **b** Baseline (*t* = 0) and maximal cAMP responses from **a** for HEK293G-WT and A2A-overexpressing HEK293G cells. *p < 0.05 (*p* = 0.037); **p < 0.01 (*p* = 0.002, *p* = 0.002 or *p* = 0.008 (left to right); One-way ANOVA with Tukey’s multiple comparison test). **c** The cAMP response to mechanical stimulus in HEK293G cells transiently transfected with the HiBiT-A_2A_ receptor with and without 2-hour pre-treatment with the selective A_2A_ antagonist SCH-58261 (10 µM). **d** The maximal cAMP responses seen in HEK293G cells transiently transfected with the HiBiT-A_2A_ receptor following 2-h pre-treatment with adenosine deaminase (2 U/ml), SCH-58261 (10 µM) or vehicle. **p < 0.01 (*p* = 0.003, peak A_2A_ versus peak A_2A_ + SCH-58261; *p* = 0.0095, peak A_2A_ + ADA versus peak A_2A_ + SCH-58261; One way ANOVA with Tukey’s multiple comparison test).

Overexpression of the A_2A_ receptor caused a dramatic increase in the cAMP response to mechanical stimulation of loading the assay plate into the PheraStar plate reader (Fig. 8a, b). Pre-treatment of cells overexpressing A_2A_ with the selective A_2a_ antagonist SCH-58261 dramatically reduced the cAMP response to plate movement (Fig. 8c, d). There was a small significant elevation in basal responses at time zero with A_2A_-receptor overexpression (Fig. 8b), but the main effect was on the peak response (compared to the response in un-transfected HEK293G cells (Fig. 8b)). Pre-treatment of cells with adenosine deaminase (ADA, 2 U/ml), did not significantly alter the peak cAMP response to plate movement in cells overexpressing A_2A_ (Fig. 8a, d), therefore suggesting that receptor activation is due to mechanical stimulation and not ligand binding.

## Discussion

The conformational equilibrium that exists between R and R* in the cubic ternary complex model of GPCR activation (Fig. 1a) explains the concept of constitutive receptor activity whereby basal activity can be observed in the absence of agonists in cells overexpressing native or constitutively active mutant β_2_ARs^16–18^. It also explains how ligands such as ICI-118551 can act as inverse agonists to convert active receptors (R*) into inactive conformational states (R)^19,21–23^. This is usually a consequence of the higher affinity of inverse agonists for the inactive R state of the receptor^21^. Consistent with this cubic ternary complex model, single-molecule studies of the β_2_AR have shown that individual β_2_AR molecules can undergo spontaneous transitions between R and R*^24^ and that the inverse agonist ICI-118,551 increases the frequency of deactivation (R* to R) transitions^24^. Structural studies of the β_2_AR have confirmed many features of the cubic ternary complex^11,25–29^. What is less clear is the extent to which agonist-independent constitutive receptor activity normally occurs and the potential therapeutic use of inverse agonists in reducing the elevated basal responses that result.

In the case of virally encoded GPCRs that are markedly over-expressed following viral infection, the resulting constitutive receptor activity is to be expected and a clear therapeutic role for inverse agonists to reduce the elevated basal responses is evident. A good example of this is US28 which is a chemokine GPCR encoded by the human cytomegalovirus that plays a role in cancer progression^48,49^. In its ligand-free apo-state, US28 signals constitutively^49^. However, for natively expressed human GPCRs, the role of constitutive receptor activity and inverse agonists is less clear. Some evidence is available that suggests that the local microenvironment of a receptor might regulate constitutive receptor activity. For example, different phospholipids can alter the equilibrium between the active (R*) and inactive (R) states of the receptor^29^. Furthermore, the presence of extracellular β_2_AR-associated glycan chains has been proposed as a mechanism by which β_2_ARs can be activated by pathogens (e.g. *Meningococcal* pili) pulling on these extracellular glycan chains and eliciting mechanical stimulation of the β_2_AR^6,33,34^. This effectively increases the proportion of R* conformations in the absence of added agonists. However, in this case, *Meningococcal* pili activate the β_2_AR to produce an active conformation that activates Gi and β-arrestin without any effect on Gs^6,33,34^. In the present study, we have directly investigated the potential for mechanical stimulation of β_2_ARs to enhance basal Gs-mediated responses that are susceptible to inhibition by inverse agonists.

The results obtained here strongly suggest that the transition from R to R* can be significantly enhanced by mechanical stimulation of the cells and furthermore indicate that the expression level of the β_2_AR determines the final size of the mechano-sensitive cAMP signalling response obtained. Thus, recombinant over-expression of the β_2_AR markedly enhanced the mechanical cAMP response obtained even though the initial basal responses at time zero were quite similar to those obtained in native HEK293G cells that express endogenous β_2_ARs at extremely low levels^36,38^. The mechanical stimulus generated here was a consequence of the rapid linear movement when plates were loaded into the plate reader. Manual loading of the plate into the reader did reduce the size of this mechanical response (Supplementary Fig. 8). These data confirm that the basal response obtained was enhanced by mechanical stimulation rather than being a sole consequence of inherent ongoing constitutive receptor activity. This mechano-sensitive response was not dependent upon ligand-induced receptor stimulation since the phenomenon was still observed in a ligand-binding deficient D113A mutant of the β_2_AR^26,39,40^, albeit to a lower extent than seen with the wild-type β_2_AR. This latter finding may indicate that mutation at position D113 does alter the way that changes in receptor conformation lead to functional coupling to Gs proteins or suggest that catecholamines trapped in the ECM can be released by mechano-stimulation. Additional NanoBRET binding and functional studies confirmed that the D113A mutant β_2_AR was not able to bind a fluorescent analogue of ICI-118551 or respond functionally to isoprenaline stimulation.

Propranolol, ICI-118551, carvedilol and carazolol were all able to reverse the enhancement of constitutive receptor activity induced by mechano-stimulation. Figure 3 and Supplementary Fig. 2 illustrate their different inverse agonist efficacies and kinetic profiles. Thus, ICI-118551 was the most efficacious inverse agonist producing the largest decrease in response, particularly after a period of preincubation prior to the mechano-sensitive stimulus. When the mechano-stimulation was initiated immediately prior to inverse agonist addition (Fig. 3), then the kinetic properties of each inverse agonist can be observed where an overshoot of the cAMP response was observed before sufficient inverse agonist bound to the β_2_AR in order to attenuate the final response obtained. This effect is most clearly observed in Fig. 5b, where ICI-118551 was added 30 min after the initial mechano-stimulation.

To investigate the role of extracellular β_2_AR-associated glycan chains in the mechano-sensitive cAMP responses observed in the present study, we mutated the asparagine residues on the N-terminus (N6A and N15A) and second extracellular loop (N187A) of the β_2_AR^30–32^ to alanine residues. Previous work has shown that mechano-stimulation of the β_2_AR can be achieved with beads coated with lectin that recognise sialic residues on the β_2_AR^33^. Mutation of these N-glycosylation residues markedly reduced the basal GloSensor^TM^ responses of over-expressed recombinant β_2_ARs to that observed in HEK293G cells endogenously expressing the wild-type β_2_AR. The residual response in HiBiT-β_2_AR-N6A-N15A-N187A mutant cells and native HEK293G cells was not sensitive to inhibition by 1 μM ICI-118551 suggesting that other endogenously expressed Gs-coupled GPCRs might contribute to the small residual mechano-sensitive basal responses observed. We were also able to confirm that the mechano-stimulation observed in the TS-SNAP-β_2_AR cell line was not a consequence of the presence of the N-terminal 19.4 kDa SNAP-tag. Thus, a large mechano-stimulation was still observed in HiBiT-β_2_AR cells expressing only the 1.3 kDa eleven amino acid HiBiT tag^42^ on the N-terminus of the β_2_AR and this was not enhanced when the full-length N-terminal nanoluciferase was reconstituted by the addition of the purified 18.1 kDa LgBiT polypeptide^42^ prior to monitoring mechano-sensitive GloSensor^TM^ responses.

To gain some insight into the potential for other GPCRs to elicit a mechano-sensitive GloSensor^TM^ cAMP response in HEK293G cells, we investigated the role of A_2A_ adenosine receptors which are also endogenously expressed in HEK293G cells^47^. Over-expression of HiBiT-tagged human A_2A_-receptors in this cell line led to a similar large mechano-stimulation of GloSensor^TM^ cAMP response that was markedly reduced by the A_2A_-receptor antagonist SCH-58261 acting as an inverse agonist. It is well established that ATP can be released by mechanical forces acting on cultured cells^43,44^ and this can be rapidly metabolised by ectonucleotidases^45,46^ to adenosine. However, this mechano-sensitive response produced by recombinant A_2A_-receptors did not appear to be secondary to adenosine-induced receptor stimulation since it was unaffected by pre-treatment with adenosine deaminase. These data suggest that the mechano-stimulation observed is a consequence of direct activation of the A_2A_-receptor in a similar manner to that observed with the β_2_AR. From this perspective, it is interesting that the A_2A_ possesses an N-glycosylation site (N154) in extracellular loop 2 (ECL2: UniProt P29274) that may have a similar function to N187 in ECL2 of the β_2_AR.

**Fig. 9:**
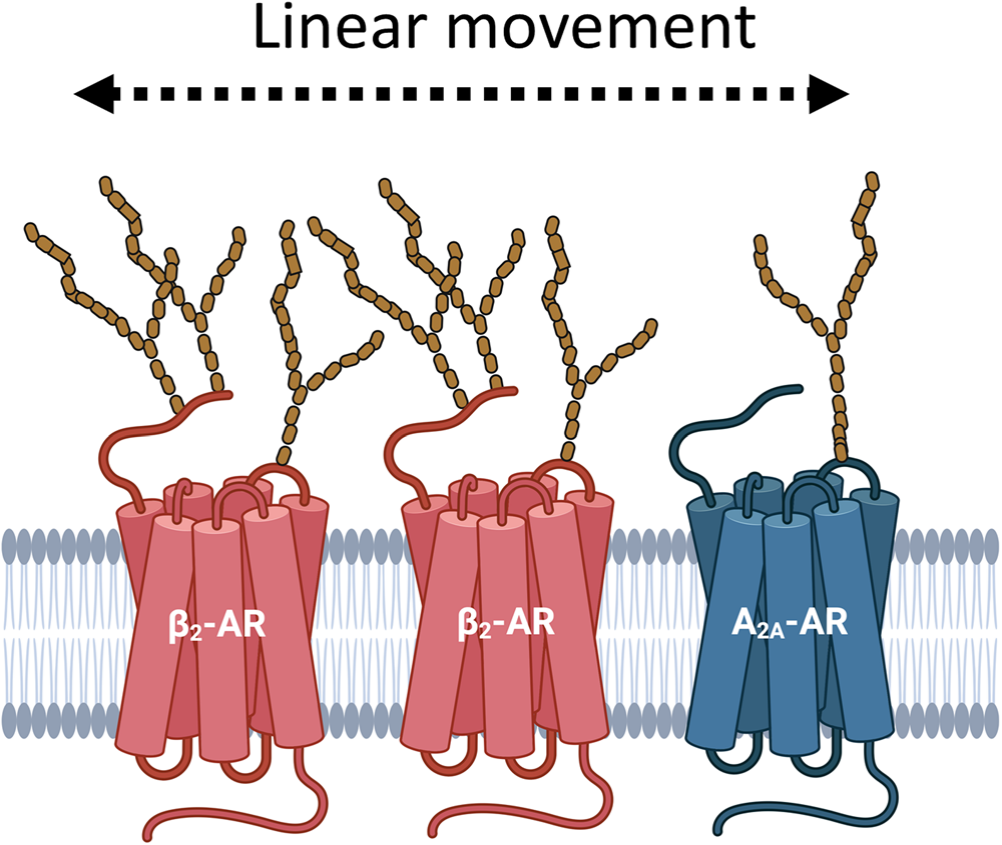
Schematic showing the potential impact of mechanical stimulation (linear movement) on extracellular N-glycan chains attached to asparagine residues on the N-terminus or second extracellular loops of the β_2_AR and adenosine A_2A_ receptor. The figure was created with Biorender.com.

The fact that rapid linear movement (caused when plates were loaded into the plate reader) was sufficient to trigger the mechano-sensitive response, whereas short linear or orbital oscillatory movements of the plate were not, suggests that a high shear stress on the extracellular domains of the β_2_AR is required^50^. In keeping with this argument, high shear stress (15 dyn cm^−1^) or equivalent stretching can lead to activation and down-regulation of the angiotensin II type 1 receptor, whereas lower oscillatory or low shear stress (3 dyn cm^−1^) produces an upregulation of this receptor^50^. Interestingly, β_2_AR is present on both vascular endothelial^5^ and bronchial epithelial cells^51^ where shear stress and stretching under normal physiological conditions may produce sufficient linear mechanical stimulation to activate receptor-mediated Gs protein signalling cascades (Fig. 9).

A number of cell surface receptors, including VEGFR2 and several members of the class A family of GPCRs, have been shown previously to be involved in ligand-independent mechano-sensing^50,52–55^. In the case of VEGFR2, a complex of proteins at cell–cell junctions, consisting of PECAM-1, VE-cadherin and VEGFR2, has been shown to be important for endothelial blood flow sensing^53,56^. Similar roles for endothelial cell mechanosensitive Gq-coupled GPCRs in flow-mediated vasodilation have also been proposed^50,52,54,57^. The results obtained in the present study suggest that Gs-coupled β_2_ARs and adenosine A_2A_ receptors must be added to the list of GPCRs that can mediate ligand-independent mechano-sensitive signalling. Furthermore, our data confirms that inverse agonists can attenuate the mechano-sensitive signalling response. For the β_2_AR, this involves extracellular glycan chains that presumably detect shear stress or interact with other proteins within the local environment of the receptors (e.g. within the extracellular matrix) to facilitate ligand-independent force sensing. The fact that these mechano-sensitive responses are sensitive to inhibition by inverse agonists should provide insights into the potential therapeutic roles of β_2_AR and A_2A_-receptor inverse agonists in regulating vascular tone^58–60^, cancer immunotherapy^61^ and preventing metastasis^62^.

## Methods

### Chemicals and reagents

The cAMP GloSensor^TM^ Human Embryonic Kidney 293 (HEK293G) cell line and GloSensor^TM^ cAMP reagent were purchased from Promega (Madison, WI, USA). The pcDNA4/TO plasmid containing the human β_2_AR with N-terminal Twin-Strep affinity purification tag and SNAP-tag (TS-SNAP-β_2_AR) construct has been described previously^37^. The DNA insert fragments (HiBiT-β_2_AR_N6A_N15A, HiBiT-β_2_AR_N6A_N15A_N187A, HiBiT-β_2_AR_D113A, and HiBiT-A_2A_) were purchased from Twist Bioscience (South San Francisco, CA, USA). Oligonucleotide primers were obtained from Sigma-Aldrich (Gillingham, UK). The Nano-Glo^®^ Luciferase Assay System, Nano-Glo^®^ HiBiT Lytic Detection System, restriction endonucleases KpnI and XbaI, FuGENE^TM^ HD were purchased from Promega (Madison, WI, USA). Gibson assembly master mix and Q5 polymerase were from New England Biolabs (Hitchin, UK). Isoprenaline hydrochloride (isoprenaline), salmeterol, propranolol, (±)-1-[2,3-(dihydro-7-methyl-1*H*-inden-4-yl)oxy]-3-[(1-methylethyl)amino]-2-butanol hydrochloride (ICI-118551), bisoprolol, carazolol, ethidium bromide, 4-(2-hydroxyethyl)-1-piperazineethanesulfonic acid (HEPES), sodium pyruvate, sodium chloride (NaCl), magnesium sulfate (MgSO_4_), Dulbecco’s modified Eagle medium (DMEM), phosphate buffered saline (PBS), trypsin-EDTA (0.25% w/v), foetal calf serum (FCS), poly-d-lysine, and adenosine were all purchased from Sigma-Aldrich (Gillingham, UK). Forskolin, formoterol, SCH-58261, and salbutamol hemisulfate were obtained from Tocris Bioscience (Bristol, UK). Polyethylenimine (PEI) transfection reagent was purchased from Polysciences (Warrington, PA, USA). Carvedilol was obtained from ACROS Organics (Geel, Belgium). Adenosine Deaminase (from calf intestine) was from Roche (Basel, Switzerland). G418 was purchased from Gibco (Loughborough, UK). The synthesis of ICI 118,551-βAla-βAla-BODIPY-X-630/650 has been described previously ^36^. Any other chemicals used were from Sigma-Aldrich (Gillingham, UK).

### Molecular biology

The pcDNA3.1 (+) Neo construct encoding HiBiT-β_2_AR was generated using the approach first described by White et al.^41^. Briefly, HiBiT-GSSG was ligated into the NLuc-β_2_AR plasmid construct^63^ to replace Nanoluciferase using the restriction enzymes KpnI and BamHI and the complementary oligonucleotides:

5’-CATG**GTGAGCGGCTGGCGGCTGTTCAAGAAGATTAGC**gggagctctggcggctcgagcggtG-3’ and 5’-GATCCaccgctcgagccgccagagctccc**GCTAATCTTCTTGAACAGCCGCCAGCCGCTCAC**CATGGTAC -3’.

The bold upper-case letters show the sequence encoding for the eleven amino acid HiBiT and the lower-case letters show the sequence encoding for the GSSGGSSG linker.

Four DNA insert fragments were also purchased from Twist Bioscience (South San Francisco, CA, USA), which comprised nucleotide sequences encoding the human β_2_AR with an N-terminal HiBiT tag (connected by a linker consisting of the following amino acid sequence: GSSGGSSGGS) and several mutations within the β_2_AR sequence, namely either a double mutation of the asparagine residues Asn6 and Asn15 to alanine residues (Ala) (HiBiT-β_2_AR_N6A_N15A fragment) or a triple mutation including the same two N-terminal asparagine residues in addition to another asparagine residue Asn187, residing on ECL2, also to alanine (HiBiT-β_2_AR_N6A_N15A_N187A fragment). Furthermore, DNA encoding β_2_AR with an N-terminal HiBiT tag and a mutation of Asp113 to alanine (HiBiT-β_2_AR D113A) and the A_2A_ receptor with an N-terminal HiBiT tag was purchased in the same format. These DNA fragments were flanked by KpnI (5’ end) and XbaI (3’ end) restriction sites for digestion, as well as Twist Universal Adaptors (forward 5’CAATCCGCCCTCACTACAACCG-3’ and reverse 5’TCCCTCATCGACGCCAGAGTAG-3’) distal to the restriction sites on each end to enable use of the same primers for each fragment during PCR amplification of the DNA. PCR-amplified DNA was then ligated into pcDNA3.1+ using the KpnI and XbaI restriction sites. DNA sequences were confirmed by whole vector sequencing performed by Plasmidsaurus (Eugene, OR, USA). The translated amino acid sequences of these plasmids are provided in Supplementary Tables 2 and 3. It should also be noted that, in all of the HiBiT plasmids, the start codon of the GPCR has been switched to leucine (Leu).

For the generation of β_2_AR C-terminal-NLuc-expressing plasmids, pcDNA3.1+ Zeo containing NanoLuciferase with an M-L mutation of the start codon and preceding ELE linker was PCR amplified using the primers forward 5’ gagctcgagctggtcttc 3’ and reverse 5’ catggtggatccgagctc 3’. DNA encoding either WT, D113A mutant or N6A, N15A, N187A mutant β_2_AR was PCR amplified using the primers described in Supplementary Table 4. Gibson assembly^64^ was then performed using Gibson assembly mastermix (NEB) according to the manufacturer’s protocols. The resultant plasmids, β_2_AR-ELE-NLuc in pcDNA3.1+ Zeo, were all verified by whole plasmid sequencing.

To generate a Nb80-mCherry plasmid, our previously described pSIN-Nb80-GFP plasmid^65^ was PCR amplified with primers forward 5’-tggacgagctgtacaagtaagaattcggatcctagataactgagc-3’ and reverse 5’-cctcgcccttgctcaccatGGTactagtgaccggtaTgtg-3’. DNA encoding the red fluorescent protein mCherry was amplified using primers forward 5’-acataccggtcactagtaccatggtgagcaagggcgagga-3’ and reverse 5’-gttatctaggatccgaattcttacttgtacagctcgtccatgc-3’. Gibson assembly was performed with the resultant PCR products, and the plasmid pSIN-Nb80-mCherry generated encoding for Nb80 with a c-terminus mCherry fusion, separated by an IPVTST linker. The plasmid was verified by whole plasmid sequencing.

### Generation of a high expression TS-SNAP-tag β_2_AR cell line

A confluent T75 flask of HEK293G cells was split and transferred to a new T75 flask at high density (1:3 split) in 20 ml media. After preincubation for twenty minutes at room temperature, 500 µl Opti-mem containing 3% polyethylenimine (PEI) transfection reagent and 5 µg pcDNA4/TO TS-SNAP-β_2_AR DNA (3:1 PEI:DNA ratio) were added to the T75 flask containing the cells and incubated at 37 °C and 5% CO_2_ for 48 h to allow transfection of the cells with the DNA. Transfected cells were then selected for 3 weeks with 50 µg/ml zeocin (selection for the pcDNA4/TO plasmid) and 100 µg/ml hygromycin B (selection for the GloSensor^TM^ plasmid). Dilution cloning was then used to obtain a stable cell line expressing both the TS-SNAP-β_2_AR DNA and the GloSensor^TM^ biosensor.

### Generation of HiBiT-β_2_AR cell lines

HEK293G cells were transfected in T25 flasks while at 60% confluency. Cells were transfected with 8.8 µg of DNA encoding either HiBiT-β_2_AR or HiBiT-β_2_AR_D113A utilising FugeneHD at a 3:1 ratio. Twenty-four hours following transfection, cells were selected for 3 weeks with 1 mg/ml G418 and 100 µg/ml hygromycin B. Cells lines were used as high-expression mixed populations without dilution cloning.

### Generation of an Nb80-mCherry cell line

HEK 293G cells were transfected in a T25 flask while at ~60% confluency with 8.8 µg pSIN-Nb80-mCherry plasmid DNA utilising FugeneHD at a 3:1 ratio. Twenty-four hours following transfection, cells were selected for two weeks with 10 µg/ml Blasticidin. Cells were sorted via FACS to select single-cell clones stably expressing the Nb80-mCherry fusion. Single-cell clones were expanded to give clonal cell populations. A single clonal cell line was used for all experiments.

### Cell culture

HEK293G (wild-type and β_2_AR-overexpressing) cells stably expressing the cAMP GloSensor^TM^ (20F) biosensor were maintained in DMEM supplemented with 10% FCS at 37 °C and 5% CO_2_. For assays, cells were seeded at 30,000 or 40,000 cells/well (dependent on cell growth rate) into white-walled, clear-bottomed 96-well plates (655098; Greiner Bio-One, Stonehouse, UK), coated with 10 µg/ml poly-d-lysine, in 100 µl media/well. The seeded plates were then incubated at 37 °C and 5% CO_2_ for 24 h prior to assay.

### Transient transfections of HiBiT-tagged wild-type and mutant constructs

For assays involving transient overexpression of β_2_AR and A_2A_ constructs contained within the pcDNA3.1(+) plasmids (HiBiT-β_2_AR-WT, HiBiT-β_2_AR_N6A_N15A, HiBiT-β_2_AR_N6A_N15A_N187A, HiBiT-A_2A_ or pcDNA3.1(+) empty vector control), HEK293G cells were first seeded at 500,000 cells/well into clear six-well plates with 2 ml media/well and incubated at 37 °C and 5% CO_2_ for 6 h or 24 h. Cells were transfected with 1 µg DNA/well in 100 µl Optimem and 3 µl FuGENE^TM^ HD and incubated at 37 °C and 5% CO_2_ for 18 h. Cells were then dislodged from the six-well plates with 500 µl 1X trypsin-EDTA and seeded at 40,000 cells/well into white-walled, clear-bottomed 96-well plates (Greiner Bio-One, Stonehouse, UK), coated with 10 µg/ml poly-d-lysine, in 100 µl media/well. The seeded plates were then incubated in DMEM supplemented with 10% FCS at 37 °C and 5% CO_2_ for 24 h prior to assay.

### cAMP GloSensor^TM^ luminescence assay

The cAMP GloSensor^TM^ luminescence assay was performed according to the manufacturer’s instructions (Promega, Madison, WI, USA). Briefly, after 24 h incubation at 37 °C and 5% CO_2_ after cell plating, media was aspirated from each well of the 96-well plate. Cells were incubated in 50 µl HEPES buffered saline solution (HBSS; 2 mM sodium pyruvate, 145 mM NaCl, 10 mM D-glucose, 5 mM KCl, 1 mM MgSO_4_·7H_2_O, 10 mM HEPES, 1.3 mM CaCl_2_, 1.5 mM NaHCO_3_ in double-distilled water, pH 7.45) containing 3% GloSensor^TM^ cAMP reagent at 37 °C for 2 h. An opaque white seal was placed on the back of the plate before reading. For agonist studies, an initial baseline luminescence read was made at time zero, the plate was then removed from the plate-reader, and a further 50 µl HBSS containing agonist (2× final concentration) or HBSS (vehicle control) was added. Luminescence was measured on an open channel (gain of 3600) immediately after these additions and then continuously over 60 min, reading each well once every minute by a PHERAstar FSX microplate reader (BMG Labtech, Offenburg, Germany). Increases in luminescence are indicative of intracellular cAMP accumulation, thus the temporal changes in relative cytosolic cAMP concentration were measured upon agonist or vehicle addition. All conditions were performed in 3–6 replicates within each plate.

For assays with an extended baseline, luminescence was measured on the PheraSTAR FSX microplate reader (BMG Labtech, Offenburg, Germany) at a gain of 3600 for 15 min following either automated loading into the PheraSTAR FSX or manual loading by hand to reduce baseline mechano-stimulation. The plate was removed from the microplate reader and 50 µl HBSS alone, containing a final concentration of 1 µM ICI 118,551 or no addition, was added to cells and luminescence was measured continuously for a further 60 min. Conditions were performed in triplicate, and 5–7 individual experiments were conducted.

### HiBiT-LgBiT complementation assay

This assay was used to determine relative cell surface expression of transiently transfected β_2_AR constructs containing N-terminal HiBiT tags (HiBiT-β_2_ARwt, HiBiT-β_2_AR_N6A_N15A, HiBiT-β_2_AR_N6A_N15A_N187A or HiBiT-D113A-β_2_AR) or pcDNA3.1(+) in HEK293G cells. The 11 amino acid HiBiT tag is a high affinity (*K*_D_: 700 pM) peptide that complements the large subunit LgBiT (18.1 kDa) to form the complete nanoluciferase enzyme^42^. Upon complementation, the complete enzyme becomes active and, in the presence of the furimazine substrate, produces luminescence which can be measured to determine relative HiBiT-tagged protein expression^42,66^. After 24 h incubation of HEK293G cells expressing HiBiT-tagged β_2_ARs in 96-well plates at 37 °C and 5% CO_2_ growth media was aspirated and cells were then incubated with 0.2% purified LgBiT protein and 0.2% Nano-Glo^®^ luciferase substrate furimazine in 100 µl HBSS and incubated at 37 °C for 5 min (to allow for equilibration of LgBiT and the furimazine-based substrate with the HiBiT tagged at the extracellular surface of the receptor) before a single luminescence measurement (open channel, 2000 gain) of each well was taken by a PHERAstar FSX microplate reader. As LgBiT is membrane impermeable, this method provides an estimate of the cell surface expression of the HiBiT-tagged protein^67^.

### HiBiT-LgBiT recomplementation prior to mechano-stimulation

HEK293G cells stably expressing wild-type HiBiT/β_2_ or the D113A mutant HiBiT/β_2_ were seeded into a 96-well plate at a density of 40,000 cells per well and incubated overnight at 37 °C and 5% CO_2_. Culture media was removed, and cells were incubated with 50 µl HBSS containing 3% GloSensor^TM^ cAMP reagent alone or with 0.2% purified LgBiT protein at 37 °C for 2 h. A white seal was placed on the back of the plate before reading. Baseline luminescence was measured on the PheraSTAR FSX microplate reader at a gain of 3600 for 15 min. The plate was removed from the microplate reader for ~60 s, then placed back into the microplate reader and luminescence was measured continuously for a further 60 min. Conditions were performed in triplicate and six individual experiments were conducted.

### Fluorescent ligand binding studies with fluorescent ICI-118551

HEK293G cells stably expressing wild-type HiBiT/β_2_, HiBiT-D113A-β_2_AR or HiBiT-β_2_AR_N6A_N15A_N187A were seeded into a 96-well plate at a density of 40,000 cells per well and incubated overnight at 37 °C and 5% CO_2_. Culture media was removed and replaced with 50 µl HBSS supplemented with 0.01% Bovine Serum Albumin (BSA) alone or containing ICI-118,551 (final concentration 50 μM) and incubated at 37 °C for 20 min. Following this incubation, HBSS with 0.01% BSA containing various concentrations (5–200 nM final concentration) of ICI-118,551-βAla-βAla-BODIPY-X-630/650^36^ was added to cells and the plate incubated at 37 °C for a further 40 min. HBSS with 0.01% BSA containing a final concentration of 0.2% furimazine and 0.2% purified LgBiT protein was subsequently added to cells and incubated at 37 °C for 5 min to allow for the signal to equilibrate. BRET signal was measured on the PheraSTAR FSX microplate reader, emission filters 460 nm (80 nm band pass) and 610LP with 2800 and 3600 gain, respectively. Conditions were performed in triplicate, and five individual experiments were conducted.

### β_2_AR-NLuc–Nb80-mCherry recruitment assays

Clonal HEK293G Nb80-mCherry cells were transfected in six-well plates with 500 ng DNA encoding either WT, D113A, or N6A, N15A, N187A β_2_AR with a C-terminus Nanoluciferase tag. Twenty-four hours following transfection, cells were seeded in white-walled clear bottomed 96-well plates and incubated for a further 24  h. For experiments, growth media was aspirated and replaced with HBSS. Cells were treated with furimazine to a final concentration of 1:400 and isoprenaline (varying concentrations). 15 min following ligand addition, BRET signal was measured on the PheraSTAR FSX microplate reader using emission filters 450 nm (80 nm band pass) and 610 long pass with 1400 and 3600 gain, respectively. BRET ratios were calculated as the ratio of the 610 long pass to the 450 bandpass signal. Five independent experiments were performed, with each experimental condition measured in triplicate.

### Data analysis and statistics

Data were analysed and presented using GraphPad Prism 10 software (San Diego, CA, USA).

The Hill equation, shown in Eq. (1), was used to fit concentration–response data to a standard sigmoidal curve, where ‘*E’* represents the magnitude of response, ‘*E*_max_’ represents the maximal response magnitude, ‘[*A*]’ is the ligand concentration, ‘EC_50_’ is the half-maximal response concentration and ‘*n*’ is the Hill coefficient.

1 $$\frac{{E}}{{{E}}_{\max }}=\frac{{\left[{A}\right]}^{{n}}}{{{{{{{\rm{EC}}}}}}}_{50}^{{n}}+{\left[{A}\right]}^{{n}}}$$

Inverse agonist concentration-response data were also fit to a sigmoidal curve by using the Hill Equation, displayed in Eq.(2), where ‘*I*’ represents the magnitude of inhibition, ‘*I*_max_’ represents the maximal inhibition, ‘[*A*]’ is the ligand concentration, ‘IC_50_’ is the half-maximal inhibition concentration and ‘*n*’ is the Hill coefficient.

2 $$\frac{{I}}{{{I}}_{\max }}=\frac{{\left[{A}\right]}^{{n}}}{{{{{{{\rm{IC}}}}}}}_{50}^{{n}}+{\left[{A}\right]}^{{n}}}$$

### Statistics and reproducibility

Results are generally expressed as mean ± standard error of the mean (SEM) from five separate experiments unless otherwise stated. The number of experimental repeats ‘n’ is stated throughout. Parallel measurements were made at each time-point following the addition of HBSS in place of agonist under the same experimental conditions. Statistical analyses were performed using GraphPad Prism 10 software (San Diego, CA, USA). Statistical significance of data was tested using either unpaired, two-tailed *t*-test, one-way ANOVA, or two-way ANOVA with Tukey’s or Dunnett’s multiple comparisons test. Throughout the study, *P* < 0.05 was used as the level of significance.

### Reporting summary

Further information on research design is available in the Nature Portfolio Reporting Summary linked to this article.

### Supplementary information

Supplementary Information

Description of Additional Supplementary Files

Supplementary Data

Reporting Summary

## Data Availability

The numerical source data behind the graphs can be found in the Supplementary Data file. All other data are available from the corresponding author on reasonable request.
