# Supplementary material for: Mechano-sensitivity of β2-adrenoceptors enhances constitutive activation of cAMP generation that is inhibited by inverse agonists
Source: Commun Biol. 2024 Apr 5;7:417. doi: 10.1038/s42003-024-06128-2 (PMC10997663; doi:10.1038/s42003-024-06128-2)
Supplement: Supplementary file 1 — Supplementary Information [file 42003_2024_6128_MOESM1_ESM.pdf]

Supplementary Information.

Mechano-sensitivity of β2-adrenoceptors enhances constitutive activation of cAMP generation that is inhibited by inverse agonists.

Sean A. Cullum[1,2], Simon Platt[1,2], Natasha Dale[1,2], Oliver C. Isaac[1,2], Edward S. Wragg[1,2], Mark Soave[1,2], Dmitry B. Veprintsev[1,2], Jeanette Woolard[1,2], Laura E. Kilpatrick[2,3] and Stephen J. Hill[1,2,*]

[1] Division of Physiology, Pharmacology and Neuroscience, School of Life Sciences, University of Nottingham, Nottingham NG7 2UH, UK
[2] Centre of Membrane Proteins and Receptors, University of Birmingham and Nottingham, The Midlands, UK
[3] Division of Bimolecular Science and Medicinal Chemistry, School of Pharmacy, Biodiscovery Institute, University of Nottingham, NG7 2RD, UK

**Inverse agonist**	**Log IC_{50} (M)**	**Maximum inhibition (%)**	**Log IC_{50} (M)**	**Maximum inhibition (%)**
	t = 0	t = 0	t = -30 min	t = -30 min
Propranolol	-8.29 ± 0.08	38.99 ± 5.81	-9.31 ± 0.21	58.52 ± 9.88*
ICI-118551	-7.83 ± 0.05	25.14 ± 3.36	-8.76 ± 0.16	25.09 ± 3.66
Carvedilol	-7.55 ± 0.08	41.10 ± 4.32	-8.76 ± 0.35	53.98 ± 10.56
Carazolol	-8.47 ± 0.05	40.56 ± 2.84	-9.11 ± 0.58	41.96 ± 7.68

Supplementary Table 1: Inverse agonist log IC_{50} and maximal inhibition at 10 □M inverse agonist determined for inhibition of peak basal cAMP GloSensor^{TM} in HEK293G cells overexpressing recombinant TS-SNAP-β_2AR. Inverse agonists were either added at time zero or pre-incubated for 30 min prior to measurement of luminescence with the PheraStar. Values are mean ± SEM from five independent experiments. In both cases, an initial measurement of luminescence was made at time zero, the plate was then removed from the PheraStar followed by immediate addition of inverse agonist (if not preincubated) and the plate was then returned to the PheraStar for continued measurements of luminescence every minute. * ($p<0.05$; $p=0.037$) compared to corresponding ICI-118551 data (One-way ANOVA with Dunnett's multiple comparison test).

DNA insert	Amino acid sequence
HiBiT- β$_2$AR	MVSGWRLFKKISGSSGGSSGGS**L**GQPGAGSAFLLAPARSHAPDHDVTQ QRDEVWVVGMGIVMSLIVLAIVFGNVLVITAIAKFERLQTVTNYFITSLACA DLVMGLAVVPFGAAHILMKMWTFGNFWCEFWTSIDVLCVTASIETLCVIAV DRYFAITSPFKYQSLLTKNKARVIILMVWIVSGLTSFLPIQMHWYRATHQEA INCYANETCCDFFTNQAYAIASSIVSFYVPLVIMVFVYSRVFQEAKRQLQKI DKSEGRFHVQNLSQVEQDGRTGHGLRRSSKFCLKEHKALKTLGIIMGTFT LCWLPFFIVNIVHVIQDNLIRKEVYILLNWIGYVNSGFNPLIYCRSPDFRIAF QELLCLRRSSLKAYGNGYSSNGNTGEQSGYHVEQEKENKLLCEDLPGTE DFVGHQGTVPSDNIDSQGRNCSTNDSLL
HiBiT- β$_2$AR_N6A_ N15A	MVSGWRLFKKISGSSGGSSGGS**L**GQPGAGSAFLLAPARSHAPDHDVTQ QRDEVWVVGMGIVMSLIVLAIVFGNVLVITAIAKFERLQTVTNYFITSLACA DLVMGLAVVPFGAAHILMKMWTFGNFWCEFWTSIDVLCVTASIETLCVIAV DRYFAITSPFKYQSLLTKNKARVIILMVWIVSGLTSFLPIQMHWYRATHQEA INCYANETCCDFFTNQAYAIASSIVSFYVPLVIMVFVYSRVFQEAKRQLQKI DKSEGRFHVQNLSQVEQDGRTGHGLRRSSKFCLKEHKALKTLGIIMGTFT LCWLPFFIVNIVHVIQDNLIRKEVYILLNWIGYVNSGFNPLIYCRSPDFRIAF QELLCLRRSSLKAYGNGYSSNGNTGEQSGYHVEQEKENKLLCEDLPGTE DFVGHQGTVPSDNIDSQGRNCSTNDSLL
HiBiT- β$_2$AR_N6A_ N15A_N187 A	MVSGWRLFKKISGSSGGSSGGS**L**GQPGAGSAFLLAPARSHAPDHDVTQ QRDEVWVVGMGIVMSLIVLAIVFGNVLVITAIAKFERLQTVTNYFITSLACA DLVMGLAVVPFGAAHILMKMWTFGNFWCEFWTSIDVLCVTASIETLCVIAV DRYFAITSPFKYQSLLTKNKARVIILMVWIVSGLTSFLPIQMHWYRATHQEA INCYAAETCCDFFTNQAYAIASSIVSFYVPLVIMVFVYSRVFQEAKRQLQKI DKSEGRFHVQNLSQVEQDGRTGHGLRRSSKFCLKEHKALKTLGIIMGTFT LCWLPFFIVNIVHVIQDNLIRKEVYILLNWIGYVNSGFNPLIYCRSPDFRIAF QELLCLRRSSLKAYGNGYSSNGNTGEQSGYHVEQEKENKLLCEDLPGTE DFVGHQGTVPSDNIDSQGRNCSTNDSLL
HiBiT- β$_2$AR_D113 A	MVSGWRLFKKISGSSGGSSGGS**L**GQPGAGSAFLLAPARSHAPDHDVTQ QRDEVWVVGMGIVMSLIVLAIVFGNVLVITAIAKFERLQTVTNYFITSLACA DLVMGLAVVPFGAAHILMKMWTFGNFWCEFWTSIAVLCVTASIETLCVIAV DRYFAITSPFKYQSLLTKNKARVIILMVWIVSGLTSFLPIQMHWYRATHQEA INCYANETCCDFFTNQAYAIASSIVSFYVPLVIMVFVYSRVFQEAKRQLQKI DKSEGRFHVQNLSQVEQDGRTGHGLRRSSKFCLKEHKALKTLGIIMGTFT LCWLPFFIVNIVHVIQDNLIRKEVYILLNWIGYVNSGFNPLIYCRSPDFRIAF QELLCLRRSSLKAYGNGYSSNGNTGEQSGYHVEQEKENKLLCEDLPGTE DFVGHQGTVPSDNIDSQGRNCSTNDSLL

Supplementary Table 2: Translated amino acid sequences of the mutated HiBiT-β$_2$AR DNA insert fragments, with the HiBiT tag highlighted in blue, linker in black, the β$_2$AR in green and the mutated nucleotide bases in red. The change of the normal start codon of the β$_2$AR from M to L is also indicated as a bold purple letter.

DNA insert	Amino acid sequence
HiBiT-A_{2A}	MVSGWRLFKKISGSSGGSSGGSLPIMGSSVYITVELAIAVLAILGNVLVCWAVWLN SNLQNVTNYFVVSLAAADIAVGVLAIPFAITISTGFCAACHGCLFIACFVLVLTQSSI FSLLAIAIDRYIAIRIPLRYNGLVTGTRAKGIIAICWVLSFAIGLTPMLGWNNCGQPK EGKNHSQGCGEGQVACLFEDVVPMNYMVYFNFFACVLVPLLLMLGVYLRIFLAA RRQLKQMESQPLPGERARSTLQKEVHAAKSLAIIVGLFALCWLPLHIINCFTFFCPD CSHAPLWLMYLAIVLSHTNSVVNPFIYAYRIREFRQTFRKIIRSHVLRQQEPFKAAG TSARVLAAHGSDGEQVSLRLNGHPPGVWANGSAPHPERRPNGYALGLVSGGSAQE SQGNTGLPDVELLSHELKGVCPEPPGLDDPLAQDGAGVS

Supplementary Table 3: Translated amino acid sequences of HiBiT-A_{2A} receptor DNA insert fragments, with the HiBiT tag highlighted in blue, linker in black, the A_{2A} receptor in green.

β_2AR variant	Forward primer (5' to 3')	Reverse primer (5' to 3')
WT, D113A	ccgagctcggatccaccatggggcaacccgggaacggcag	gtgaagaccagctcgagctccagcagtgagtcatttgtactacaattcctc
N6A,N15A, N187A triple mutant	ccgagctcggatccaccatggggcaacccggggccggcag	gtgaagaccagctcgagctccagcagtgagtcatttgtactacaattcctcccttgt

Supplementary Table 4: PCR primers used to amplify DNA encoding WT, D113A mutant or, N6A, N15, N187A mutant β_2AR for Gibson assembly of β_2AR-NLuc plasmids.

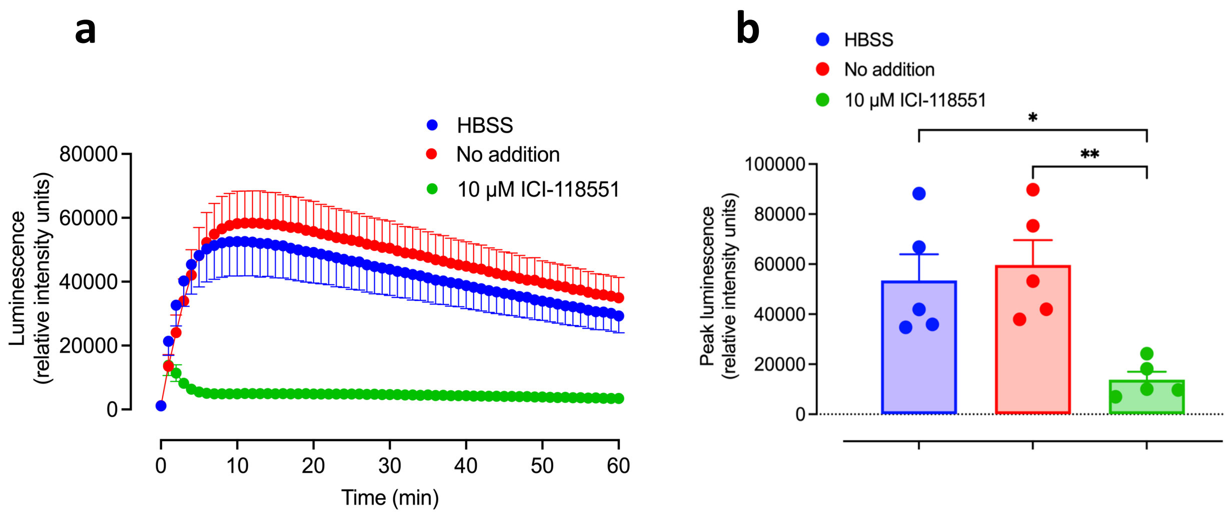

Supplementary Figure 1. (a) Time course of the basal Glosensor^{TM} response obtained following the addition of HBSS, ICI-118551 or no addition in an HEK293G clonal cell line over-expressing the transfected human TS-SNAP-β_2AR. Where appropriate, HBSS or ICI-118551 was added immediately following an initial luminescence read at time zero. The plate therefore entered the PheraStar at time zero, was then removed for addition of inverse agonist, HBSS or no addition and measurements made again at t=1 min and every minute thereafter. Values are mean ± SEM of 5 independent experiments. In each individual experiment triplicate determinations were made. (b) The effect of HBSS, ICI-118551 or no addition on the peak basal response. Values are mean ± SEM from 5 independent experiments. ** $p<0.01$ or * $p<0.05$ compared to the peak HBSS basal responses in the presence of ICI-118551 (one-way ANOVA; n=5). The no addition peak responses were not significantly different from HBSS ($p=0.87$; one-way ANOVA, n=5).

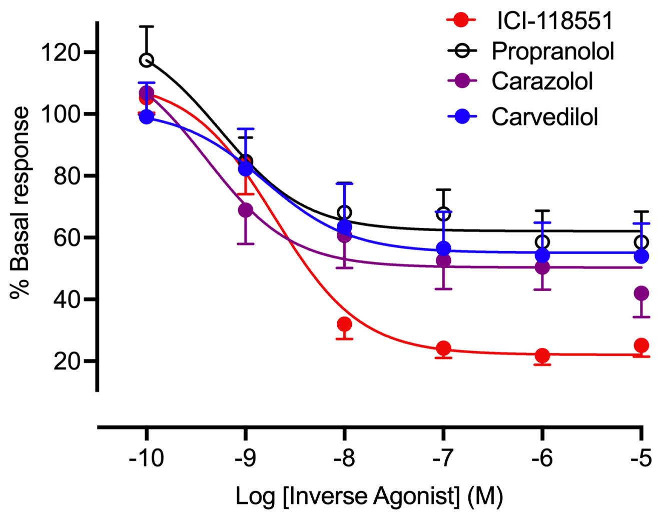

Supplementary Figure 2. Effect of 30 min pre-incubation with inverse agonists on the peak responses to HBSS obtained in an HEK293G clonal cell line over-expressing the transfected human TS-SNAP-β_2AR. Each inverse agonist was added 30 min prior to measurement of luminescence with the PheraStar. The plate was added to the PheraStar at time zero for an initial luminescence read and then removed and re-inserted before measurements were made again at t=1 min and every minute thereafter. Values are mean ± SEM of 5 independent experiments. In each individual experiment triplicate determinations were made. (e) Effect of four inverse agonists on the peak basal response to HBSS. Values are mean ± SEM from 5 independent experiments. Data are expressed as a percentage of the peak basal response obtained in each individual experiment.

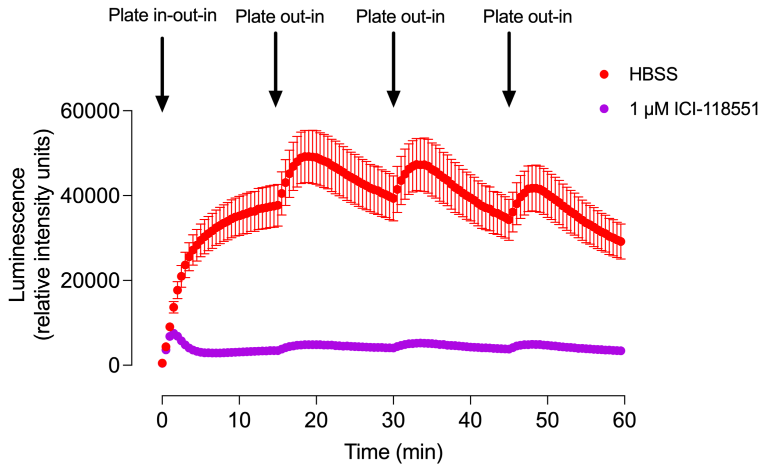

Supplementary Figure 3. Impact of mechanical stimulation on basal Glosensor^{TM} time-course responses in a clonal HEK293G cell line over expressing recombinant TS-SNAP-β_2AR conducted in the dark. In all experiments, an initial luminescence read was made at time zero before any additions. The plate was then immediately removed, HBSS or ICI-118551 (1 □M) added and then the plate was returned to the PheraStar. Measurements were then made at 1 min and every min for 60 min in the dark. At 15 min, 30 min and 45 min the motorised stage of the PheraStar removed the plate from the instrument and then immediately returned it into the plate reader for further measurements every min. Values are mean ± SEM from 5 independent experiments. In each individual experiment triplicate determinations were made.

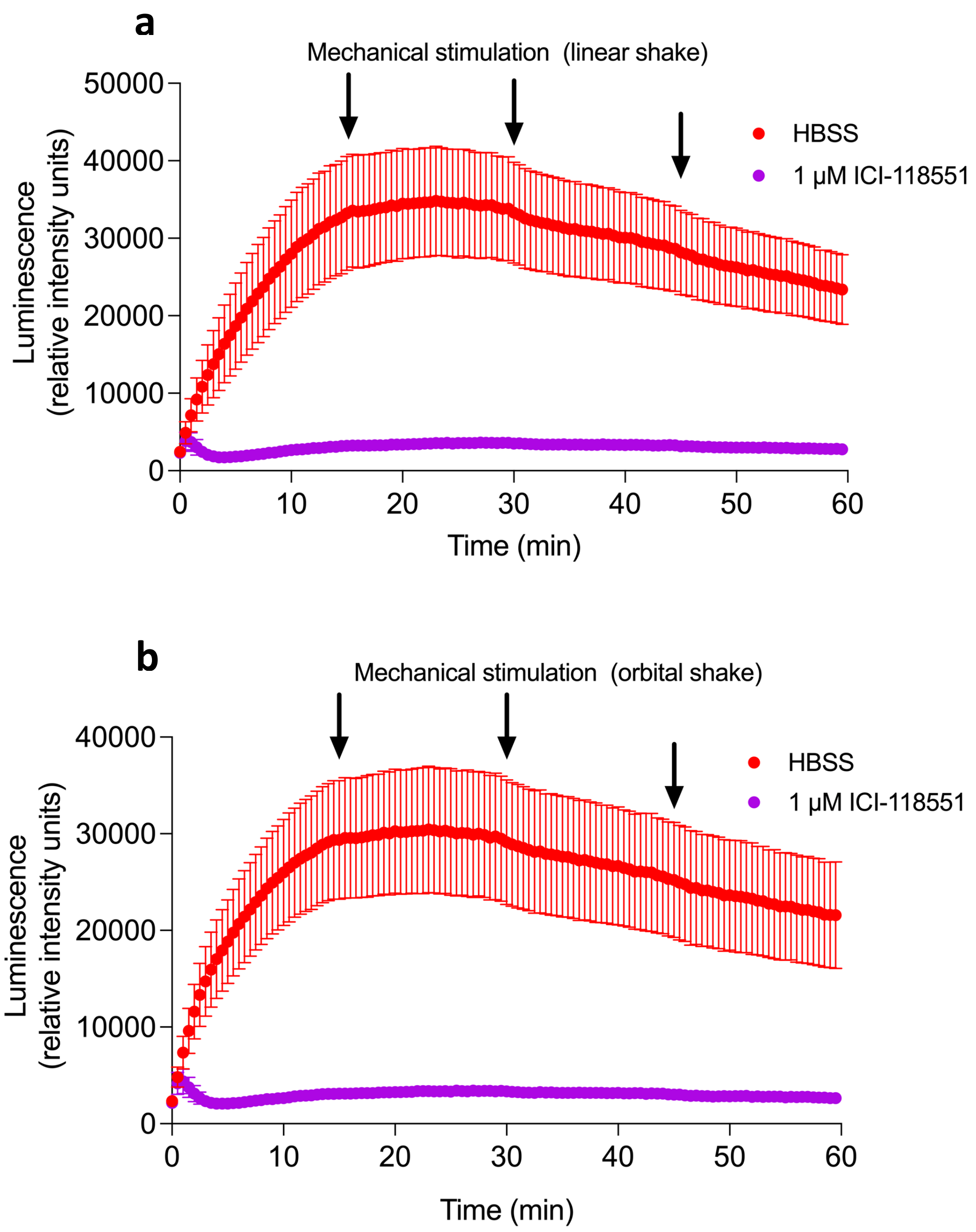

Supplementary Figure 4. Impact of linear or orbital shakes on basal Glosensor^{TM} responses in a clonal HEK293G cell line over expressing recombinant TS-SNAP-β_2AR. In these experiments, an initial luminescence read was made at time zero before any additions. The plate was then immediately removed, HBSS or ICI-118551 (1 □M) added and then the plate was returned to the PheraStar. Measurements were then made at 1 min and every min for 60 min. At 15, 30 min and 45 min the motorised stage of the PheraStar exerted a 5s (a) linear shake (100 rpm) or (b) orbital shake. Values are mean ± SEM from 5 independent experiments. In each individual experiment triplicate determinations were made.

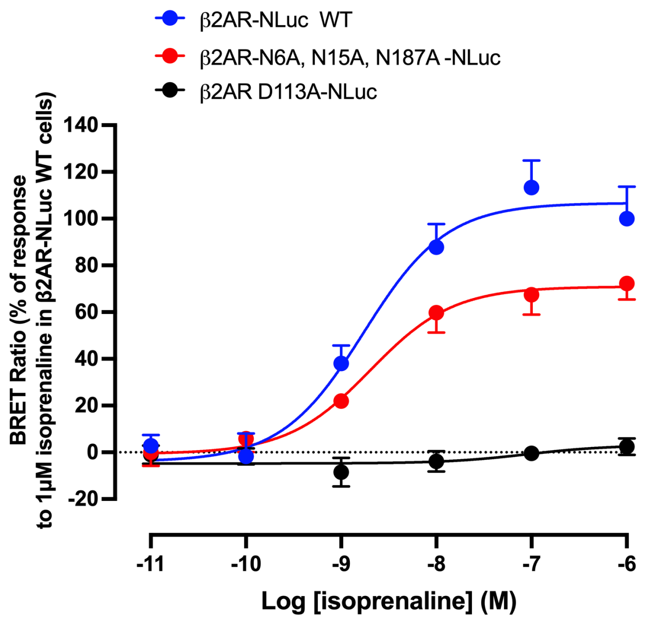

Supplementary Figure 5. Effect of isoprenaline on the interaction of C-terminal nanoluciferase (NLuc)-tagged receptors with mCherry-tagged nanobody-80 (Nb-80) monitored using NanoBRET. Experiments were performed following transient transfection of wild-type (WT) β_2AR-NLuc, β_2AR-N6A-N15A-N187A-NLuc or β_2AR-D113A-NLuc into HEK293G cells stably expressing mCherry-Nb80. Values are mean ± SEM of 5 independent experiments. In each individual experiment triplicate determinations were made. Data are expressed as a percentage of the response to 1 □M isoprenaline monitored in cells expressing the wild-type -β_2AR-NLuc receptor obtained in each individual experiment.

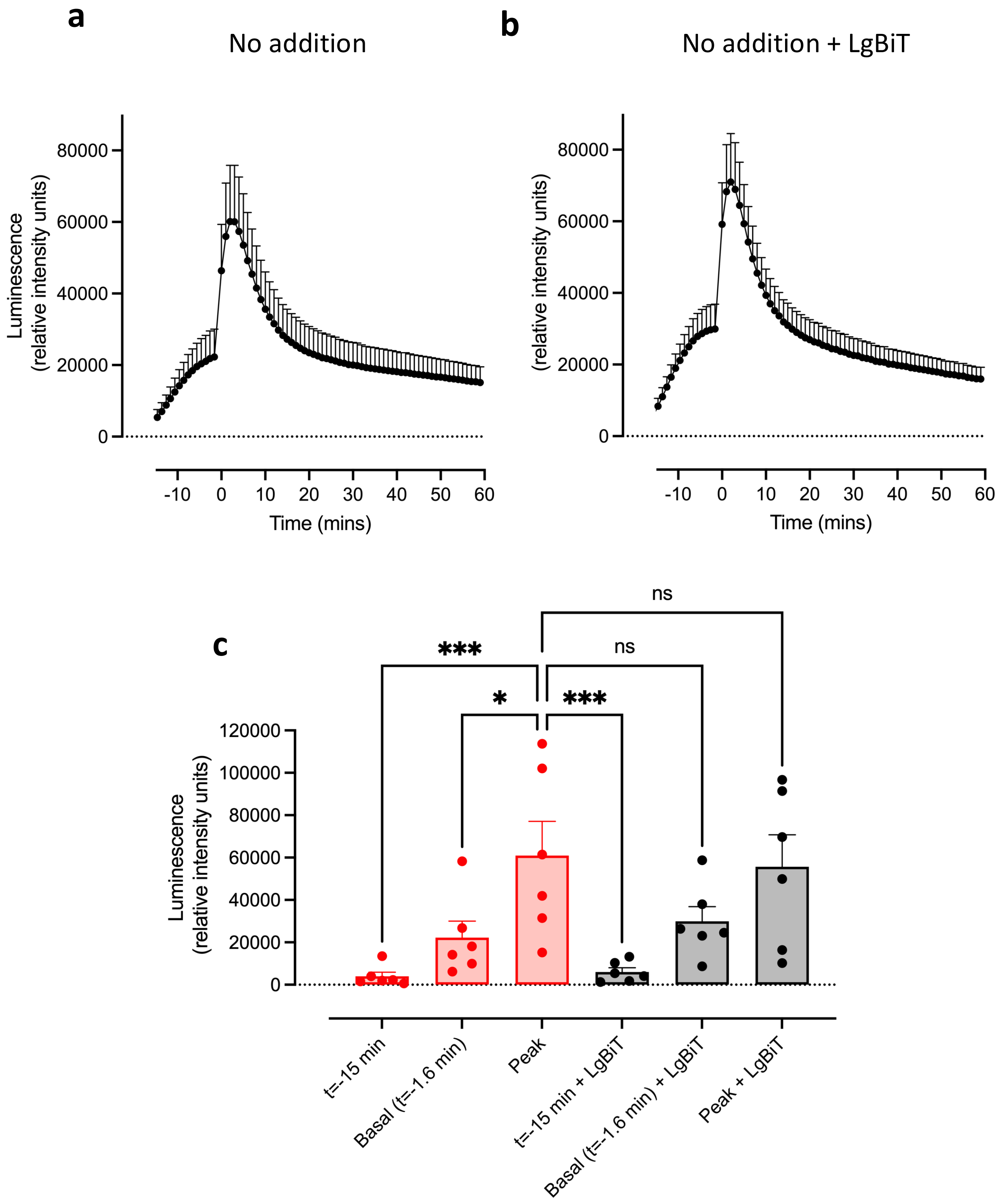

Supplementary Figure 6. Impact of LgBiT addition on the basal Glosensor responses obtained in a stable HEK293G cell line expressing HiBiT-β_2AR. (a,b) Time course of the basal Glosensor^{TM} responses in HEK293G cells expressing HiBiT-β_2AR in the absence (a) or presence of LgBiT to reconstitute full-length N-terminal nanoluciferase. In both (a) and (b), the plate was placed in the PheraStar at t=-15 min, the plate was then removed at time zero and immediately returned to the PheraStar with no additions. Where appropriate, 0.2%

purified LgBiT was added two hours before the start of the experiment. Values are mean ± SEM from 6 independent experiments. (c) Comparison of mean responses obtained at t= -15 min, t= -1.6 min (basal) and the peak of the responses obtained in HEK293G cells expressing HiBiT-β_2AR in the absence or presence of 10ng LgBiT to reconstitute the full-length N-terminal nanoluciferase tag. *** $p < 0.001$ or * $p<0.05$ (two-way ANOVA with Sidak's multiple comparison test). Peak versus t -15min (p=0.0006); peak versus basal (t=-1.6 min) (p= 0.024); peak versus t= -15 min +LgBiT (p=0.0009); peak versus basal (t=-1.6 min) + LgBiT (ns; p=0.097); peak versus peak + LgBiT (ns; p=0.99).

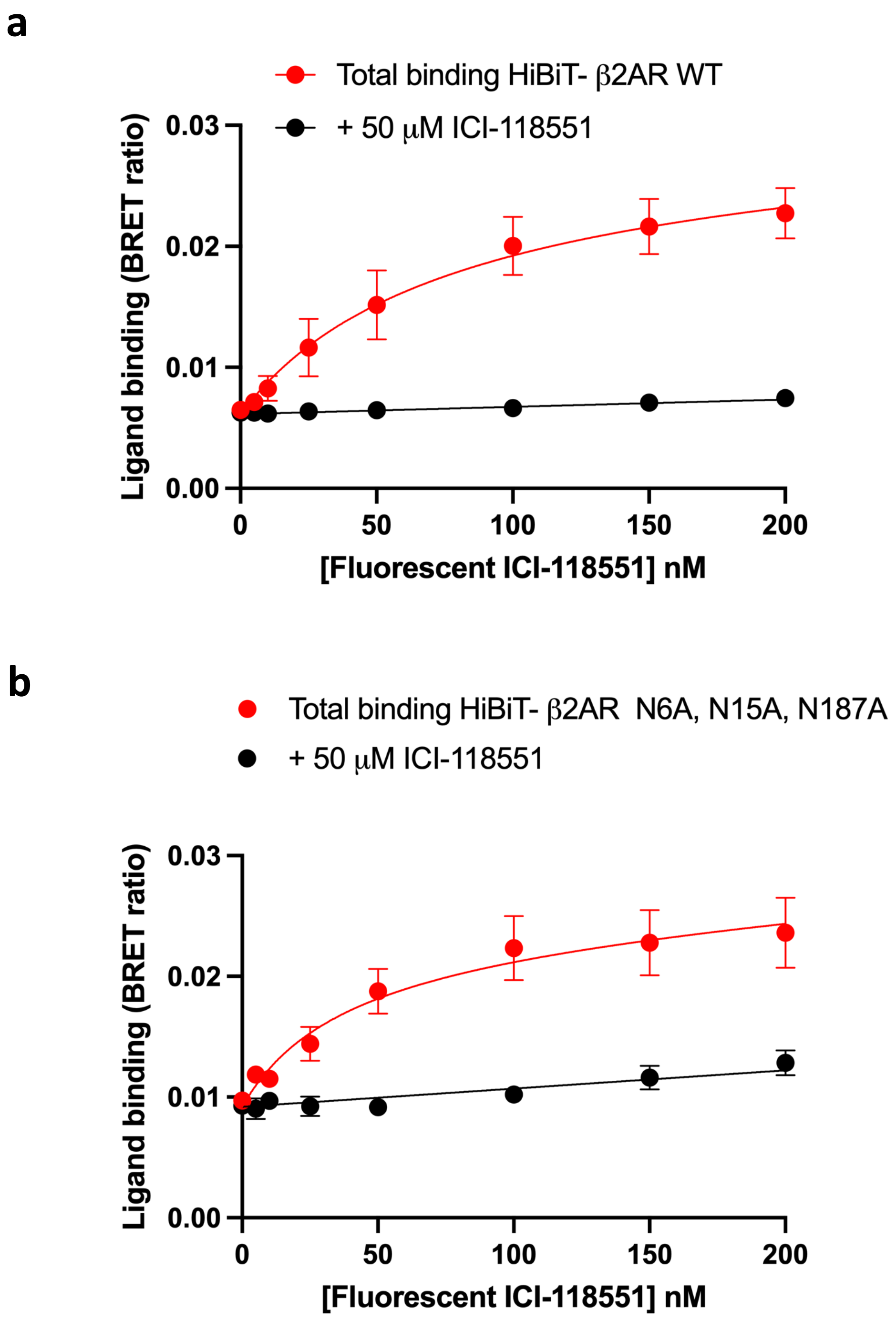

Supplementary Figure 7. Total and non-specific binding of ICI-118,551-βAla-βAla-BODIPY-X-630/650 (fluorescent ICI-118551) to HEK293G cells expressing (a) wild-type (WT) HiBiT-β_2AR or (b) HiBiT- β_2AR-N6A-N15A-N187A. Binding was determined following re-complementation of full length nanoluciferase with the addition of 0.2% purified LgBiT. Non-specific binding was determined in the presence of 50 μM ICI-118551. K_D values are (a) 74.7nM for binding to WT HiBiT-β2AR and (b) 38.1nM for binding to HiBiT- β_2AR-N6A-N15A-N187A. Values are mean ± SEM from 5 independent experiments.

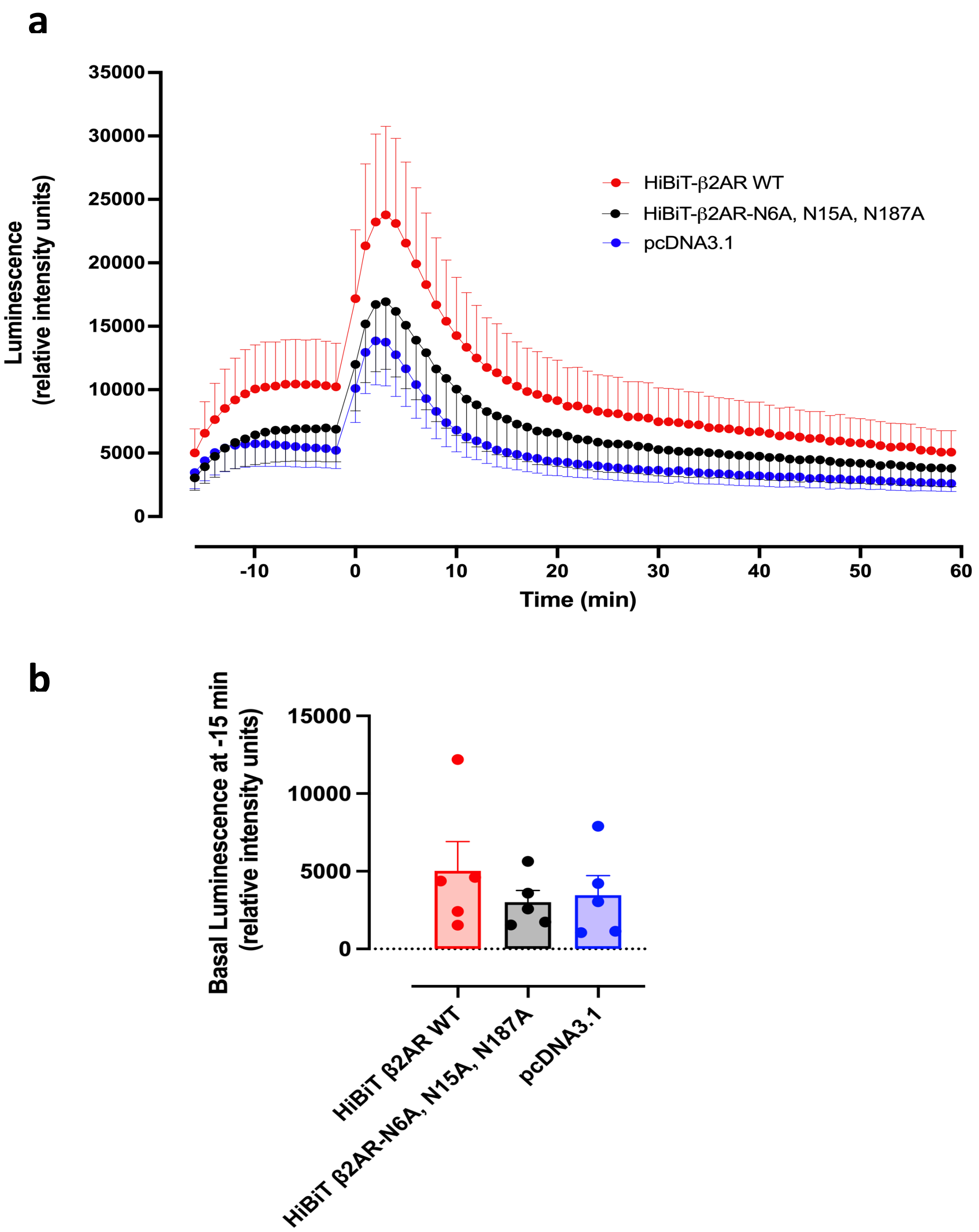

Supplementary Figure 8. The effect of manual plate loading on Glosensor^{TM} responses obtained in HEKG cells transiently transfected with wild-type (WT) HiBiT-β2AR, HiBiT- β_2AR-N6A-N15A-N187A or pcDNA3. (a) Time course of Glosensor^{TM} response following manual plate loading at t=-15 min followed by automated plate movement out and in of the platereader at t=0 min. (b) Individual Glosensor^{TM} responses at t=-15 min. There was no significant difference in luminescence at t=-15min (two-way ANOVA with Tukey's multiple comparison test; p=0.38 and p= 0.32 for HiBiT-β2AR wild-type versus pcDNA3 or HiBiT-β_2AR-N6A-N15A-N187A respectively). Values are mean ± SEM from 5 independent experiments.
